# A comprehensive model to understand and assess the motivational background of video game use: The Gaming Motivation Inventory (GMI)

**DOI:** 10.1556/2006.2022.00048

**Published:** 2022-08-08

**Authors:** Orsolya Király, Joël Billieux, Daniel L. King, Róbert Urbán, Patrik Koncz, Eszter Polgár, Zsolt Demetrovics

**Affiliations:** 1 Institute of Psychology, ELTE Eötvös Loránd University, Budapest, Hungary; 2 Institute of Psychology, University of Lausanne, Lausanne, Switzerland; 3 Centre for Excessive Gambling, Addiction Medicine, Lausanne University Hospitals, Lausanne, Switzerland; 4 College of Education, Psychology, & Social Work, Flinders University, Adelaide, Australia; 5 Doctoral School of Psychology, ELTE Eötvös Loránd University, Budapest, Hungary; 6 Centre of Excellence in Responsible Gaming, University of Gibraltar, Gibraltar, Gibraltar

**Keywords:** video games, motivation, psychometrics, surveys and questionnaires, gaming disorder, behavior, addictive

## Abstract

**Background and aims:**

The popularity of video gaming has generated significant interest in research methods to examine motivations for gaming. Current measures of gaming motives are limited by lack of scope and/or their applicability to specific game genres only. We aimed to create a comprehensive motivation inventory applicable to any gaming genre and to evaluate its psychometric properties in a large sample of highly engaged video gamers.

**Methods:**

Stage 1 of this project involved a systematic review that generated the items for the Gaming Motivation Inventory (GMI). Stages 2–4 involved an evaluation of the psychometric properties of the GMI. A sample of 14,740 video gamers (89.3% male; mean age 24.1 years) were recruited via an online survey promoted by a popular gaming magazine.

**Results:**

In Stage 2, twenty-six gaming motives were identified, which clustered into six higher-order dimensions (Mastery, Immersion/Escapism, Competition, Stimulation, Social, Habit/Boredom). In Stage 3, construct validity of the six higher-order motives was assessed by associations with gaming-related, personality, and psychological variables. In Stage 4, the relationships between motives and depression symptoms and gaming disorder symptoms were explored. Although gaming motives had weak associations with gaming genres, they were moderately related to variables such as competitiveness, sociability, and positive and negative affect. Gaming disorder symptoms were directly predicted by depression symptoms and indirectly via Immersion/Escapism, Habit/Boredom, and Competition motives.

**Discussion and conclusions:**

These findings support the notion that motives are one of the primary causes of gaming behavior and play an important role in predicting its problematic nature. The GMI is a psychometrically valid tool that will be useful for gaining insights into factors underlying gaming behaviors.

The popularity of video gaming is continuously growing in all regions of the world (Statista, 2021) and its appeal extends to many different demographic groups (Entertainment Software Association, 2021). Because society spends a considerable amount of time and money on video games, it is important to understand this activity, including its benefits and risks. Video games help people relax, connect, and be entertained (Jones, Scholes, Johnson, Katsikitis, & Carras, 2014). Furthermore, they have great potential in several areas, such as education (Janarthanan, 2012), improving cognitive functioning (Wang et al., 2016), and other skills (e.g., hand-eye coordination; Gupta et al., 2021). However, gaming can be implicated in cases of toxic online behavior and cyberbullying (Kordyaka, Jahn, & Niehaves, 2020; Kwak, Blackburn, & Han, 2015; Paul, 2018), and, for some vulnerable individuals, it can become uncontrolled and associated with addiction-like symptoms and functional impairment (Castro-Calvo et al., 2021; Király et al., 2018; Rumpf et al., 2018).

Motivation is the psychological force that activates and maintains goal-directed thought and behavior (Wasserman & Wasserman, 2020); the study of gaming motives therefore has utility in understanding the popularity of video games. The attraction of video games rests in their ability to pull people in and keep them engaged for long periods because such games are highly rewarding. They are designed to motivate players in numerous ways to both strengthen engagement and appeal to a large customer base. Although early video games such as Pong and Tetris mostly appealed to a player's need for competence, video games today usually aim to satisfy a wide range of psychological needs (Przybylski, Rigby, & Ryan, 2010).

Studying gaming motives is crucial in exploring the line between healthy and problematic engagement. For example, research on alcohol use and alcohol use disorder has consistently found that drinking motivation is of high importance in determining decisions about whether to drink or not (Cooper, 1994; Kuntsche, Knibbe, Engels, & Gmel, 2007), and motivation explains up to 50% of the variance in adolescent alcohol use (Kuntsche, 2007). Building on these findings, research exploring the role of motives in various problematic and non-problematic use has emerged (e.g., video gaming, gambling, cannabis and new psychoactive substance use, various online activities such as TV series watching and compulsive sexual behavior; e.g., Benschop et al., 2020; Flayelle et al., 2019; Koós, Fuss, Klein, Demetrovics, & Bőthe, 2022; Simons, Correia, Carey, & Borsari, 1998; Stewart & Zack, 2008).

Problematic or addictive engagement in video games was recognized as an official diagnosis in the *International Classification of Diseases and Related Health Problems*, 11th ed., in 2019 under the name gaming disorder (GD; Billieux, Stein, Castro-Calvo, Higushi, & King, 2021; Reed et al., 2022). It refers to a condition manifested by a persistent or recurrent gaming behavior – over a period of at least 12 months – characterized by an impaired control over gaming, increasing priority given to gaming over other activities to the extent that gaming takes precedence over other interests and daily activities and continuation of gaming despite the occurrence of negative consequences. The behavior pattern is of sufficient severity to result in significant impairment in personal, family, social, educational, occupational or other important areas of functioning (World Health Organization, 2019; code 6C51).

Studies have shown that some motives (e.g., escapism: playing video games to avoid everyday problems and difficulties) are associated with GD (Billieux et al., 2015; Kwon, Chung, & Lee, 2011) and also constitute mediators between psychiatric symptoms and GD (Ballabio et al., 2017; Király et al., 2015). One explanation is that players who struggle with psychopathological symptoms such as depression or anxiety are prone to escaping in games to avoid their problems and relieve negative affect, thus promoting overinvolvement and the development and maintenance of GD (Di Blasi et al., 2019; Kardefelt-Winther, 2014a). Consequently, studying gaming motives is crucial, from both theoretical and applied perspectives (e.g., prevention, intervention), for understanding healthy and passionate gaming, as well as problematic engagement.

Research in video gaming motives has a relatively long history. The earliest and most cited empirical model and measurement scale was created among massively multiplayer online role-playing game (MMORPG) players in the early 2000s. Yee (2006) built his model based on Bartle's early work on player types in multi-user dungeons, that is, text-based virtual environments (Bartle, 1996), and created a motivational model with 10 different motives that clustered into three overarching motivational components: (a) Achievement: advancement, mechanics, and competition; (b) Social: socializing, relationship, and teamwork; and (c) Immersion: discovery, role playing, customization, and escapism. Although Yee's model is comprehensive and frequently used in research, it was developed primarily for MMORPG players and many of its items apply to MMORPGs (e.g., “How important is it to you that your character's armor/outfit matches in color and style?”). Yee's market research company, Quantic Foundry (https://quanticfoundry.com/), proposed a model comprising 12 distinct motives belonging to six higher-order motivation clusters (Action: destruction and excitement; Social: competition and community; Mastery: challenge and strategy; Achievement: completion and power; Immersion: fantasy and story; Creativity: design and discovery; Yee, 2015b). This model is robust, tested on more than 500,000 gamers worldwide. However, it is not openly accessible to the scientific community due to copyright protection. Another issue is that the model removed the escapism motivation, which has clinical relevance due to its moderate-to-strong association with GD (Bányai, Griffith, Demetrovics, & Király, 2019; Melodia, Canale, & Griffiths, 2022). Game designers may be more interested in motives that are related to game characteristics that can be modified (e.g., fantasy motive: features that provide authenticity to the game world, such as non-player characters), whereas the escapism motive is more about the player than the game.

Another established motivational scale developed from a large sample of online game players of several different genres is the Motives for Online Gaming Questionnaire (MOGQ; Demetrovics et al., 2011), comprising seven gaming motives: recreation, social, competition, skill development, escape, fantasy, and coping. After a series of exploratory factor analyses (EFAs) and confirmatory factor analyses (CFAs), a distinction was made between escape and coping motives, the latter referring to playing games to reduce stress and aggression or to improve mood. The most recent scale is the Videogaming Motives Questionnaire (López-Fernández, Mezquita, Griffiths, Ortet, & Ibáñez, 2020), which was developed by building on the previous instruments. It comprises eight motives: recreation, social interaction, coping, violent reward, fantasy, cognitive development, customization, and competition.

Although these instruments were developed by generating large item pools and using a factor analytical approach to arrive at a final model, other instruments were developed mainly from a theoretical perspective. For instance, the Player Experience of Need Satisfaction scale (Ryan, Rigby, & Przybylski, 2006) and the Gaming Motivation Scale (Lafrenière, Verner-Filion, & Vallerand, 2012) were both based on self-determination theory, which posits that people have three basic psychological needs: competence, autonomy, and relatedness to others. Satisfaction of these needs contributes to well-being and is associated with autonomous motivation, whereas frustration of these needs contributes to ill-being and is related to lower quality and highly controlled forms of motivation (Ryan, Ryan, Di Domenico, & Deci, 2019). The Player Experience of Need Satisfaction scale assesses perceived in-game autonomy, competence, and relatedness, and the Gaming Motivation Scale assesses intrinsic (i.e., when someone engages in an activity solely because he or she enjoys it and gets personal satisfaction from it) and extrinsic motivation (i.e., when someone does something in order to gain an external reward), as well as amotivation (i.e., the relative lack of motivation) related to video gaming.

Existing motivational scales have several limitations. First, although they cover a number of different motives (see Table 1 in López-Fernández et al., 2020), none of them cover all motives and some of the comprehensive scales lack the escapism motivation, which has the highest clinical relevance because of its moderate-to-strong association with GD. Second, many of the instruments are genre specific and therefore are not generic instruments that can be used independently of the game genre. Because game genres are constantly evolving, instruments need to be as generic as possible to avoid becoming rapidly outdated with the emergence of new game genres. Third, as a result of fast technological advancement, video games evolve rapidly and some of the scales or items become outdated because new terms and game mechanics are used.

To address the limitations of existing measurement instruments of gaming motives, we aimed in this study to create a new motivation inventory that is comprehensive (i.e., covering all motives identified in the literature) and genre neutral; that is, it can be applied to any video game genres. To achieve these objectives, we conducted a systematic literature review to identify all of the gaming motivation scales developed up to late 2019. We then selected the most popular scales, along with the motivational factors from less used scales that seemed relevant to consider, and developed a comprehensive item pool that covered all motives in the scales. We aimed to cover each gaming motive with three to five items and test the psychometric properties of the factors created. Furthermore, we assumed that numerous different motives would emerge from the systematic literature search and measurement instruments identified; therefore, we aimed to test the higher-order structure of these motives and the construct validity of the structure obtained.

## Overview of study stages

The study comprised four separate analytical stages. In Stage 1, we conducted a systematic literature review of studies in which psychometric instruments were applied to assess video gaming motives empirically. From these instruments, a comprehensive and genre-neutral item pool was generated, in which items were clustered in motivational factors derived from the previous literature on gaming motives. Data were collected from a large-scale sample of highle engaged video game players to examine (a) the psychometric properties and higher-order structure of the motivational factors (Stage 2); (b) associations of the higher-order motivational dimensions with demographic, gaming-related, personality, and psychological variables (Stage 3); and (c) associations of the motives with depression symptoms, GD symptoms, and gaming time (Stage 4). The aim in Stages 2 and 3 was to test the construct validity of the identified motives.

### Ethics

The study was approved by the Institutional Review Board of ELTE Eötvös Loránd University and was performed in line with the Helsinki Declaration.

## Method and results

### Stage 1: Systematic literature review and item pool creation

To develop a comprehensive and genre-neutral pool of items assessing gaming motives, we first ran a systematic literature review to identify studies in which gaming motives were measured empirically. We conducted a computer database search of PubMed, ScienceDirect, Web of Science, and Scopus on November 21, 2019, using the following search terms and logic: (videogame OR videogames OR video game OR video games OR videogaming OR video gaming OR computer game OR computer games OR computer gaming OR internet game OR internet games OR internet gaming OR digital game OR digital games OR digital gaming OR online game OR online games OR online gaming OR excessive gaming OR compulsive gaming OR gaming addiction OR gaming disorder OR problematic gaming OR pathological gaming) AND (motive OR motives OR motivation OR motivations OR motivational). All searches were limited to full-text papers (i.e., journal articles, book chapters, and review papers) published in English. These database search parameters yielded a total of 3,965 hits: PubMed (396 results), ScienceDirect (300 results), Scopus (1,745 results), Web of Science (1,524 results). After duplicates were deleted, 2,463 hits remained. Abstracts and full texts, where necessary, were examined and studies were selected on the basis of using a psychometric instrument to assess video gaming motives empirically. The reference lists of the papers included were also examined. In total, 163 papers were retained (see Supplemental Figure S1 in the online supplemental materials for a flowchart of the search procedure including exclusion criteria; the table comprising the selected papers can be requested from the corresponding author).

Next, the most frequently used motivational instruments were identified, along with motivational factors from less used scales that were assessed by the present authors as not identical, yet relevant to consider (e.g., the items had some incremental validity; Supplemental Table S1 in the online supplemental materials). A list of 100 items covering 27 motives was generated from these instruments and factors. Considering that these items were meant to serve as the initial item pool for the development of the Gaming Motivation Inventory (GMI), we aimed to be as comprehensive and inclusive as possible, covering motives used consistently in previous studies, as well as those proposed to address recent developments in gaming (e.g., financial motives related to earning money in video games, such as in e-sports, blockchain developments). Furthermore, we aimed to generate items that were genre-neutral, which involved modifying some items to remove specific references to certain games or features. The items followed two different formats: (a) “Why do you play video games? I play video games…” and (b) “What kind of gameplay do you prefer? I like video games that…”. Both item formats were answered on a 7-point Likert scale from 1 (*does not correspond at all*) to 7 (*it corresponds exactly*). The 100 items and the generation process are presented in detail in Supplemental Table S2 in the online supplemental materials.

### Stages 2–4: Empirical analyses using data from a gamer sample

#### Participants and procedure

An online survey was designed and administered in Qualtrics (https://www.qualtrics.com) to collect data from highly engaged video gamers (i.e., a term referring to gaming as a consistent, usually daily, routine involving regular long sessions or frequent short sessions, and which typically amount to a substantial weekly commitment that approximates part-time or full-time employment). The most popular Hungarian gaming magazine, *GameStar*, targeting the Hungarian-speaking gamer community (living in Hungary and surrounding countries such as Romania, Serbia, Slovakia, and Ukraine), promoted our survey among online readers and Facebook followers. Three advertisements containing the link to the questionnaire were posted on the magazine's website and Facebook page in March and April 2020 (a period when stay-at-home restrictions due to the COVID-19 pandemic were in force in the target countries). Paid Facebook ads were also used to reach the target audience during this period. Incentives were offered in the form of shopping vouchers (20 vouchers of 30 euros each, five vouchers of 60 euros each, and two vouchers of 300 euros each). All articles contained the link to the questionnaire, as well as a description of the aims of the survey, the cooperation between our research group and the gaming magazine, and the opportunity to win some of the prizes. We also emphasized that the results of the survey would be reported on the website of the magazine, which was done in the first half of 2021.^1^

Before starting the questionnaire, participants were informed about the aim of the study and the time necessary for completion and they were assured about anonymity and confidentiality. They provided informed consent by ticking a box if they agreed to continue and participate in the study (children 14–17 years old had to tick another box for parental permission). Email addresses were obtained from those willing to participate in the draw and used only to contact the winners.

On the basis of our previous data collection experiences with the same gaming magazine, we aimed for a sample of a minimum of 5,000 participants in approximately 4 weeks to avoid any possible timing effect and because, after the first 4 weeks, the rate of completion had considerably decreased in previous data collections. In total, 20,300 gamers started the survey. Initial data cleaning resulted in the removal of cases with severe inconsistencies, identical answers to items of longer scales, and an unreliably fast completion time (*n* = 105 in total). Because of the length of the questionnaire, we noticed a gradual attrition, in which 49.8% of the sample (*n* = 10,104) fully completed the survey and 59.7% (*n* = 12,065) had a maximum of two missing values. We decided to remove cases of participants who withdrew before answering the motivational item list. Therefore, a sample of 14,740 cases was used for the analyses. The listwise deletion method in SPSS version 25 (IBM Corp., 2017) and the full information maximum likelihood method in Mplus 8 (Muthén & Muthén, 1998–2017) were used to treat missing values.

#### Measures

Sociodemographic information (age, gender, marital status, education, current study and work status) and questions regarding gaming habits were collected. The following gaming-related variables were assessed: weekly gaming time, gaming platform, and game genres. *Average weekly gaming time* was assessed with two separate questions asking the exact hours (to one decimal) for an average weekday and weekend day. Values could be given between 0 and 12; if respondents played more than 12 h a day, they were instructed to record 12. The two variables were combined during the analysis ([hours on an average weekday × 5] + [hours on an average weekend day × 2]). *Gaming platform* use in the previous year was assessed by asking respondents to divide 100% use among four response options: (a) PC/laptop, (b) gaming console, (c) smartphone, and (d) mobile devices other than smartphones (e.g., phone, tablet, handheld console). *Game genres* were measured in a similar way for the previous year by dividing 100% use among the following response options: (a) shooters, first-person shooter, third-person shooter (e.g., Call of Duty, Counter-Strike, Overwatch), (b) battle royale (e.g., Fortnite, PUBG), (c) multiplayer online battle arena (MOBA; e.g., League of Legends, Dota 2), (d) auto chess/auto battler games (e.g., Hearthstone Battlegrounds, Teamfight Tactics, Dota Underlords), (e) open-world action-adventure (e.g., GTA series, Red Dead Redemption, Watch Dogs), (f) role-playing games (e.g., Witcher, Skyrim), (g) online role playing games (RPGs), MMORPGs (e.g., World of Warcraft, Guild Wars 2, The Elder Scrolls Online), (h) strategy games, real-time strategy, turn-based strategy (e.g., StarCraft, Hearthstone, Civilization, XCOM), (i) card games (e.g., Hearthstone, Magic: The Gathering Arena, The Elder Scrolls: Legends), (j) sport games (e.g., FIFA, Need for Speed, Madden NFL), (k) simulations (e.g., vehicle: Euro Truck Simulator, animal: Goat Simulator, life: The Sims), other (e.g., puzzle, platformer, casual games, Facebook games).

*Gaming motives* were assessed by administering the motivational item pool comprising 100 items (see Supplemental Table S2 in the online supplemental materials) to the sample. To avoid systematic missing values on the later part of the item pool because of fatigue that may have appeared due to the lengths of the inventory, we randomized the items of the two blocks for each participant individually.^2^ More specifically, we randomized items 1 to 78 of the first format “Why do you play video games? I play video games…” and then items 79–100 of the second format “What kind of gameplay do you prefer? I like video games that…” separately.

*Sociability* was assessed with five items proposed by Asendorpf and Wilpers (1998). The scale measures the preference for being with people (e.g., “I find people more stimulating than everything else”). Responses were answered on a 5-point scale from 1 (*strongly disagree*) to 5 (*strongly agree*), with one reversed item. Summarized scores ranged from 5 to 25, higher scores indicating stronger sociability. Cronbach's alpha was 0.82 in the present sample.

*Competitiveness* was assessed with three items from the nine-item Enjoyment of Competition subscale of the Revised Competitiveness Index (Harris & Houston, 2010; Houston, Harris, McIntire, & Francis, 2002). The items were as follows: (i) “I like competition,” (ii) “I am a competitive individual,” and (iii) “I find competitive situations unpleasant” (reversed item). Items were selected by taking into consideration their content and factor loadings in the original study. We decided to use only three items because of the high semantic similarity of the original nine items. Responses were given on a 5-point scale from 1 (*strongly disagree*) to 5 (*strongly agree*). Summarized scores ranged from 3 to 15, higher scores indicating a more competitive nature. Cronbach's alpha in the present sample was 0.84.

*Sensation seeking* is the tendency to enjoy and pursue activities that are stimulating or exciting and the willingness to try new and unconventional experiences. It was assessed with the four-item subscale of the short version of the UPPS-P Impulsive Behavior Scale (Billieux et al., 2012; Zsila, Bőthe, Demetrovics, Billieux, & Orosz, 2020). Responses were obtained on a 4-point scale (1 = strongly agree, 2 = agree somewhat, 3 = disagree somewhat, 4 = strongly disagree). All items were reversed during the analyses; therefore, the summed total scores ranged from 4 to 16, higher scores being indicative of stronger sensation seeking tendencies. Cronbach's alpha was 0.80 in the present sample.

*Self-esteem* was measured with the five positive items of the Rosenberg Self-Esteem Scale (Rosenberg, 1965). These items were selected to reduce the length of the questionnaire and because they cluster in a methodological factor that has a high correlation with the entire scale. Responses were provided on a 4-point scale from 1 (*strongly disagree*) to 4 (*strongly agree*). Summarized scores ranged from 5 to 20, higher scores indicating higher self-esteem. Internal consistency on the present sample was 0.87.

*Self-esteem when playing video games* was assessed with a modified version of the five positive items of the Rosenberg Self-Esteem Scale (Rosenberg, 1965). Items were complemented with the statement “When I play games, …” and respondents were asked to think about their gaming activity when answering the questions. Internal consistency on the present sample was also 0.87.

*Positive and negative affect trait version* was measured with the 20-item Positive and Negative Affect Schedule (PANAS; Watson, Clark, & Tellegen, 1988). PANAS comprises 20 words describing mood (e.g., interested, distressed, upset, strong) and has two factors, positive and negative affect (10 items each), which are highly uncorrelated with each other. Items were rated on a 5-point scale (1 = very slightly or not at all, 2 = a little, 3 = moderately, 4 = quite a bit, 5 = extremely) and respondents were asked about mood/characteristics in general (trait type). Scores on both subscales ranged from 10 to 50, with higher scores indicating higher positive or negative affect. Both scales had high internal consistencies in the present sample, α_PANAS positive_ = 0.87, α_PANAS negative_ = 0.86.

*Perceived stress* was measured for the previous 3 months with the short four-item version of the Perceived Stress Scale (Cohen, 1986; Cohen & Williamson, 1988), which assesses how uncontrollable and overloaded respondents find their lives to be and the degree to which situations in their life is appraised as stressful. Responses were obtained on a 5-point scale (1 = never, 2 = almost never, 3 = sometimes, 4 = often, 5 = very often). Summarized scores ranged from 5 to 20, with higher scores indicating higher perceived stress. Cronbach's alpha was 0.77 in the present sample.

*GD symptoms* were assessed with the Ten-Item Internet Gaming Disorder Test (IGDT-10; Király et al., 2019; Király, Sleczka, et al., 2017). The IGDT-10 assesses internet gaming disorder (IGD) criteria in the previous 12 months, as proposed in the *Diagnostic and Statistical Manual of Mental Disorders* (5th ed.; *DSM-5*; American Psychiatric Association, 2013). Items referred to video gaming in general, not only internet gaming. Responses were recorded on a 3-point scale (never, sometimes, often) and dichotomized (never and sometimes were coded as “no,” often was coded as “yes”) during the analysis to match the categorical nature of the *DSM-5*. The last IGD criterion (“Has jeopardized or lost a significant relationship, job, or educational or career opportunity because of participation in Internet games”) was operationalized with two items to avoid having double-barreled questions. These two items were merged during the analysis in such a way that an “often” response given to any of the two items meant a “yes” for the merged criterion. Furthermore, in this study, we decided to exclude Item 8 of the IGDT-10 that assesses “escapism or mood relief” to avoid overlap with the Immersion/Escapism higher-order motive when assessing the association of the two variables (see Giardina, Di Blasi, et al., 2021, for a similar approach). Thus, total scores on the IGDT-10 ranged from 0 to 8, higher scores indicating more IGD symptoms. Composite reliability for the instrument comprising the eight dichotomized items was 0.88.

*Depression symptoms* were measured with the six-item version of the Center of Epidemiological Studies-Depression Scale (CES-D; Radloff, 1977), used in the European School Survey Project on Alcohol and Other Drugs (ESPAD; Hibell et al., 2009). When answering the questions, respondents were asked to think of the previous 3 months. CES-D is not designed to diagnose clinical depression, but it is a valid screening instrument to assess depressive mood and emotional suffering. The validity of the six-item version was reported in the *2007 ESPAD Report* (Hibell et al., 2009). Items were answered on a 4-point scale (*rarely or never* to *most of the time*). Scores ranged from 4 to 24, with higher scores indicating higher depressive mood level or more depression symptoms. Cronbach's alpha was 0.81 in the present sample.

#### Transparency and openness

We report how we determined our sample size, data exclusions, all manipulations, and all measures in the study, and we follow the Journal Article Reporting Standards (Kazak, 2018). All data and analysis code are available at the open science framework: https://osf.io/tfhjx/. All research materials are described in detail in the manuscript. Analyses were performed with SPSS version 25 (IBM Corp., 2017) and Mplus 8 (Muthén & Muthén, 1998–2017). This study's design and its analysis were not preregistered.

#### Descriptive statistics

The majority of the respondents in the sample were male (89.3%), with a mean age of 24.1 years (*SD* = 7.0) and a range between 14 and 75 years. Approximately half were single (48.3%), the other half being in a relationship and living either separately (22.8%) or together (28.3%). Some of the respondents were studying at the time of data collection (47.0%), and 50.6% had a full-time job. Overall, the sample comprised highly engaged video gamers, who played 27.6 h per week on average (*SD* = 14.9). Respondents divided 100% of their time between four gaming platforms that they had used in the previous year: PCs/laptops were the most used at 47.7% on average, consoles followed at 36.2%, and then smartphones at 14.7%. Gaming genres were assessed similarly. The most popular genre in the present sample was shooters (25.1%), followed by open-world action-adventure games (17.0%), RPGs (12.0%), battle royale games (10.1%), and sport games (9.4%) (Table 1).

**Table 1.** Demographics and gaming-related information of the sample

Demographics	Total sample (*N* = 14,635–14,740)^a^
Gender, male	13,157 (89.3%)
Age, years; mean (*SD*)	24.1 (7.0)
Education (number of years completed), mean (*SD*)	13.0 (2.7)
Marital status	
Single	7,105 (48.3%)
In a relationship but living separately	3,357 (22.8%)
Married/living in a partnership	4,166 (28.3%)
Divorced	75 (0.5%)
Widowed	7 (<0.1%)
Currently a student	6,927 (47.0%)
Working status	
Does not work	5,378 (36.5%)
Has a full-time job	7,449 (50.6%)
Has a part-time job	782 (5.3%)
Works on ad hoc basis	1,125 (7.6%)
Gaming time	
On an average weekday (hours)	3.3 (2.1)
On an average weekend day (hours)	5.6 (2.7)
On an average week (hours)	27.6 (14.9)
Gaming platform (respondents divided 100% use among platforms in the previous year); mean % (*SD*)	
PC/laptop	47.7 (38.1)
Console (e.g., Xbox, PS, Wii)	36.2 (36.5)
Smartphone	14.7 (18.4)
Other mobile device (e.g., tablet)	1.4 (6.5)
Gaming genre (respondents divided 100% use among genres in the previous year); mean % (*SD*)	
Shooters, FPS, TPS (e.g., Call of Duty, Counter-Strike, Overwatch)	25.1 (24.5)
Battle royale (e.g., Fortnite, PUBG)	10.1 (18.0)
MOBA (e.g., League of Legends, Dota 2)	6.8 (16.7)
Auto chess/auto battler games (e.g., Hearthstone Battlegrounds, Teamfight Tactics, Dota Underlords)	2.3 (7.4)
Open-world action-adventure (e.g., GTA series, Red Dead Redemption, Watch Dogs)	17.0 (19.6)
Role-playing games, RPGs (e.g., Witcher, Skyrim)	12.0 (18.6)
Online role-playing games, MMORPGs (e.g., World of Warcraft, Guild Wars 2, The Elder Scrolls Online)	5.4 (14.5)
Strategy games, RTS, TBS (e.g., Starcraft, Hearthstone, Civilization, XCOM)	3.7 (9.9)
Card games (e.g., Hearthstone, Magic: The Gathering Arena, The Elder Scrolls: Legends)	1.4 (5.6)
Sport games (e.g., FIFA, Need for Speed, Madden NFL)	9.4 (17.6)
Simulations (e.g., vehicle: Euro Truck Simulator, animal: Goat Simulator, life: Sims)	3.8 (10.9)
Other (e.g., puzzle, platformer, casual games, Facebook games)	3.1 (10.1)

*Note*. PC = personal computer; FPS = first-person shooter; TPS = third-person shooter; MOBA = multiplayer online battle arena; RPG = role-playing game; MMORPGs = massively multiplayer online role-playing games; RTS = real-time strategy; TBS = turn-based strategy.

^a^ Sample size for the analyses varied due to missing values.

Data collection took place in March and April 2020 when restrictions were applied in Hungary because of the first wave of the COVID-19 pandemic. To examine the effects of the lockdown situation, we administered additional questions (see Supplemental Table S3 in the online supplemental materials). According to the results, 66.4% of the sample played more in this period than before the pandemic (28.2% played much more, and 38.2% played a bit more), whereas gaming habits (gaming types played, part of the week/day when playing, motives for gaming, and gaming partners) were unchanged for 42.0% of the sample and changed only a little for 28.0%.

### Stage 2: Higher-order structure of the motivational factors

To test the psychometric properties and higher-order structure of the theoretically proposed motivational factors, we collected data from a large sample of highly engaged video game players.

#### Statistical analysis

First, psychometric properties of the 27 theoretically proposed motivational factors were examined by using CFA and checking Cronbach's alphas as internal consistency measures separately for each factor in the total sample. A Pearson-correlation was run to test the strength of the associations between the theoretically proposed factors. On the basis of these analyses, items with low factor loadings or overlapping content were removed and all items belonging to one motivational factor were parceled into one composite score for each motive. Second, the total sample was divided into three non-overlapping random subsamples. Sample 1 (*N* = 4,905) was used to perform an initial EFA of the theoretically proposed motivational factors (items were averaged). Sample 2 (*N* = 4,894) was used to conduct a separate EFA to cross-validate the factor structure found in the first analysis. Samples 1 and 2 were used to define the factor structure tested separately, with an exploratory structural equation modeling (ESEM) analysis on Sample 3 (*N* = 4,941). The two EFAs were conducted with a principal axis factoring estimation method and oblique rotation, Promax (Kappa = 4), because factors were expected to be correlated. The number of factors was determined from the eigenvalues (larger than 1.0) and theoretical interpretability of the factors.

To cross-validate the higher-order factor structure obtained from the two EFA analyses, we conducted an ESEM analysis. In contrast to CFA, where items are defined to load only on their respective factor, whereas cross-loadings are constrained to zero, in ESEM analysis, items are defined to load on their main factors, whereas cross-loadings are “targeted,” but not forced, to be as close to zero as possible with the oblique target rotation procedure (Browne, 2001). We chose to retain all motives for the ESEM analyses despite the existence of weak factor loadings and considerable cross-loadings in order to maximize the comprehensiveness of the item pool and motives they covered. The robust maximum likelihood estimator (MLR) was used because it provides standard errors and tests of model fit that are robust to the non-normality of the data. When interpreting the magnitude of the factor loadings, the following thresholds were applied: excellent above 0.71, very good between 0.63 and 0.70, good between 0.55 and 0.62, fair between 0.44 and 0.33, and poor below 0.32 (Comrey & Lee, 2013).

To evaluate the goodness of fit of measurement models (e.g., ESEM), we relied on a combination of several different goodness-of-fit indices (Brown, 2015): the comparative fit index (CFI), the Tucker-Lewis index (TLI), the root-mean-square error of approximation (RMSEA), and the standardized root-mean-square residual (SRMR). The following rough guidelines for adequate and excellent thresholds for these fit indices (Hu & Bentler, 1999; Marsh, 2007; Marsh, Hau, & Wen, 2004; Marsh, Hau, & Grayson, 2005) were applied: values greater than 0.90 and 0.95 were considered adequate and excellent, respectively, in the case of CFI and TLI, and values smaller than 0.08 and 0.06 indicated acceptable and excellent model fit, respectively, for the RMSEA and SRMR. We also report the robust chi-square (χ^2^) test of exact fit; however, this fit index tends to be oversensitive to sample size and minor model misspecifications (Byrne, 2010). Missing data in Mplus were treated with the full information maximum likelihood method (Muthén & Muthén, 1998–2017).

#### Results

First, CFAs were performed separately on each of the 27 theoretically proposed motivational factors to examine their psychometric properties. Fit indices, modification indices, factor loadings, and internal consistency measures were examined and are shown in Supplemental Table S4 in the online supplemental materials. Each of the 27 factors comprised three to five items. When only three items covered a factor, the degree of freedom was 0 and model fit was not informative. However, factor loadings were > 0.6 for all items in all these cases (see Supplemental Table S4). For factors with four or five items, fit indices did not meet the proposed thresholds in a few cases. In these cases, items with relatively low factor loadings were removed. Where modification indices indicated high error covariance between items, one or two items per factor were removed. The process for each factor is detailed in Supplemental Table S4. After “cleaning” the factors, we conducted a correlation analysis to determine the associations between the 27 motives. All correlations were < 0.7 except for Exploration and Mechanics motives (*r* = 0.744). These factors were merged, and four items were retained in the final merged factor, fitting the data well. Psychometric details related to the newly merged factor can also be seen in Supplemental Table S4. Finally, 88 items remained, parceled in 26 motives (see Appendixes A and B for the GMI). Descriptive statistics for the motives can be seen in Supplemental Table S5 in the online supplemental materials. The strength of the associations between the 26 final motives varied between *r* = 0.003 (*P* = 0.741) and *r* = 0.695 (*P* < 0.001) and can be seen in Supplemental Table S6 in the online supplemental materials.

Second, we conducted two EFAs on two non-overlapping random subsamples (i.e., Samples 1 and 2) to investigate how the 26 motivational factors cluster into higher-order motivational dimensions. Six factors had eigenvalues above 1, and structures with five, six, and seven factors were examined. The factor structure with six factors was retained because of theoretical considerations. Table 2 shows factor loadings and percentages of total variance explained for both samples.

**Table 2.** Exploratory factor analysis of the 26 motivational factors on two non-overlapping random subsamples (*n*_1_ = 4,587; *n*_2_ = 4,562)

	Mastery		Immersion/Escapism		Competition		Stimulation		Social		Habit/Boredom	
	Sample1	Sample2	Sample1	Sample2	Sample1	Sample2	Sample1	Sample2	Sample1	Sample2	Sample1	Sample2
Advancement	0.757	0.788			0.126	0.127						
Amotivation	0.178	0.153									0.728	0.717
Autonomy	0.494	0.538	0.502	0.437								
Boredom			0.170	0.173	0.136		0.173	0.177			0.403	0.410
Competence	0.178	0.181	0.297	0.286	0.584	0.606						
Competition	0.134		−0.241	−0.185	0.696	0.697	0.208	0.230				
Completion	0.835	0.828	−0.155	−0.161	0.199	0.160			−0.153	−0.123		
Coping	−0.172	−0.154	0.700	0.681			0.138	0.150				
Escape	−0.194	−0.182	0.867	0.867								0.108
Exploration + Mechanics	0.919	0.936						−0.121			0.104	
Fantasy	0.197	0.198	0.766	0.765	−0.156	−0.185						
Financial					0.361	0.405					0.104	
Game skills	0.608	0.641	−0.118	−0.155	0.508	0.476					−0.101	
Identity	0.133	0.190	0.616	0.597	0.229	0.242	−0.152	−0.175			−0.104	−0.126
Introjected regulation	−0.101		0.518	0.539	0.381	0.360			−0.115	−0.111	0.167	0.121
Recreation		0.131	0.351	0.283	−0.181	−0.237	0.149	0.210			−0.220	−0.219
Skill development	0.312	0.387	0.163		0.281	0.260			0.131	0.127	−0.163	−0.119
Social			0.160	0.175	0.262	0.280	−0.148	−0.135	0.638	0.599		
Status				0.104	0.745	0.756						
Arousal-action					0.230	0.217	0.639	0.675				
Cooperation			−0.133	−0.103					0.895	0.910		
Customization	0.307	0.382	0.138		−0.145	−0.142	0.298	0.308				0.127
Destruction			0.153	0.127	0.161	0.163	0.556	0.558			0.126	0.110
Graphics							0.559	0.538				
Story	0.392	0.412	0.251	0.218	−0.457	−0.404	0.172	0.151				
Strategy	0.423	0.371	−0.117	−0.129	−0.107				0.322	0.356		
% of total variance explained	33.23	33.30	9.13	9.19	8.32	7.99	5.44	5.57	4.27	4.21	3.96	3.99

*Note*. Factor loadings below 0.10 are not included.

In line with the eigenvalue information, the first three factors were more robust with more motives loading strongly on them, whereas the last three factors had only two to three motives loading on them. The merged motivation of exploration and mechanics, completion, advancement, and game skills loaded strongly on the first higher-order factor, whereas autonomy, strategy, story, customization, and skill development loaded weakly, and some (autonomy, story) had cross-loadings with other higher-order factors. On this basis, the first factor was named *Mastery*. The second factor was labeled *Immersion/Escapism* because escape, fantasy, coping, identity, and introjected regulation loaded strongly on it. In addition, autonomy, recreation, competence, and story had weak loadings. The third factor had status, competition, competence, and game skills loading on it strongly and several other motives loading weakly, including financial, introjected regulation, skill development, social, action-arousal, and identity, of which only financial did not have cross-loadings on other factors. The third higher-order factor was labeled *Competition*. The fourth higher-order motivational dimension was called *Stimulation* because it had arousal-action, destruction, and graphics loading on it, whereas competition, customization, and recreation had weak loadings and cross-loadings with other higher-order factors. The fifth higher-order factor had cooperation and social loading strongly on it and strategy loading weakly. Therefore, it was labelled *Social*. The last higher-order factor was called *Habit/Boredom* because it had only two motives loading on it: amotivation and boredom.

To cross-validate this higher-order factor structure, we conducted an ESEM analysis on the third random subsample (Sample 3). The model had an adequate fit to the data, χ^2^ (184) = 3,264.4, *P* < 0.001; CFI = 0.944; TLI = 0.901; RMSEA = 0.059, 90% confidence interval 0.057–0.060; SRMR = 0.021. Autonomy, game skills, introjected regulation, skill development, and strategy motives had considerable cross-loadings. Furthermore, recreation, skill development, customization, story, and strategy had low factor loadings. Correlations between the six higher-order factors ranged from −0.013 to 0.480 (Table 3), the strongest association being between Competition and Social.

**Table 3.** Exploratory structural equation modeling of the 26 motivational factors from the results of the exploratory factor analysis (*n*_3_ = 4,872)

	Factor loadings					
	Mastery	Immersion/Escapism	Competition	Stimulation	Social	Habit/Boredom
Advancement	**0.730**	0.018	0.196	0.059	−0.080	−0.011
Amotivation	0.057	−0.032	0.012	−0.049	0.033	**0.677**
Autonomy	**0.533**	**0.458**	−0.040	0.021	0.000	0.030
Boredom	−0.024	0.093	0.066	0.167	−0.013	**0.464**
Competence	0.145	**0.332**	**0.606**	0.021	0.005	−0.014
Competition	0.101	−0.183	**0.660**	0.243	0.053	0.043
Completion	**0.778**	−0.087	0.193	0.018	−0.102	0.037
Coping	−0.070	**0.631**	0.115	0.158	0.046	−0.051
Escape	−0.113	**0.783**	0.050	0.020	−0.011	0.099
Exploration + Mechanics	**0.878**	0.030	−0.014	−0.147	0.085	0.071
Fantasy	0.217	**0.735**	−0.109	0.073	−0.053	0.035
Financial	0.024	0.028	0.293	−0.111	0.140	0.127
Game skills	**0.622**	−0.093	**0.503**	0.019	0.019	−0.058
Identity	0.159	**0.620**	0.273	−0.133	0.061	−0.111
Introjected regulation	−0.075	**0.499**	**0.335**	0.002	−0.033	0.173
Recreation	0.159	0.271	−0.129	0.191	0.035	−0.237
Skill development	**0.384**	0.136	**0.306**	−0.008	0.132	−0.144
Social	0.018	0.163	0.178	−0.102	**0.694**	−0.010
Status	0.005	0.125	**0.692**	0.035	0.144	0.054
Arousal-action	0.079	−0.009	0.222	**0.644**	0.111	−0.052
Cooperation	0.009	−0.124	−0.020	0.102	**0.875**	0.005
Customization	**0.324**	0.127	−0.143	0.286	0.106	0.075
Destruction	−0.007	0.156	0.086	**0.534**	0.025	0.165
Graphics	0.090	0.015	0.004	**0.503**	−0.042	−0.165
Story	0.344	0.237	**−0.341**	0.180	0.010	−0.038
Strategy	**0.390**	−0.104	−0.113	0.093	**0.359**	0.042

Correlation between the factors					
	Mastery	Immersion/Escapism	Competition	Stimulation	Social
Immersion/Escapism	0.447***				
Competition	0.329***	0.269***			
Stimulation	0.436***	0.350***	0.124***		
Social	0.337***	0.191***	0.480***	0.276***	
Habit/Boredom	−0.223***	0.111***	0.172***	−0.013	0.047*

*Note*. Salient factor loadings (>0.30) are boldfaced.

*P < 0.05. ***P < 0.001.

### Stage 3: Associations of the higher-order motivational dimensions with demographic, gaming-related, personality and psychological variables

In Stage 3, we aimed to investigate the associations of the higher-order motivational dimensions with gaming genre, demographic, gaming-related, personality, and psychological variables to test the construct validity of the model.

#### Statistical analysis

Gaming motives were introduced in the analysis as latent variables (the six higher-order motives as defined in the ESEM analysis). A correlation analysis between the six higher-order motives and the game genres was conducted, complemented with a graphical illustration of the associations between these variables. Factor scores of the six higher-order motives obtained from the ESEM analysis (having a mean of 0 and standard deviation of 1) were rescaled with minimum-maximum normalization ranging from 0 to 100.

We performed a multiple indicators multiple causes (MIMIC) analysis with the MLR estimation method in Mplus 8 to validate the six-factor motivational structure and test the association with personality traits (sociability, competitiveness, sensation seeking) and psychological variables (self-esteem, positive and negative affect, perceived stress) on the total sample. Gender and age were introduced in the model as control variables. The MIMIC technique, a specification of SEM, was chosen because it can estimate the effect of indicators on latent variables (the six higher-order motives as defined in the ESEM analysis) at the same time when direct effects of grouping variables or other continuous variables on the latent variables are also included, and it increases the precision of estimations because of the elimination of measurement errors. In addition, the zero-order correlation matrix of the variables included in the MIMIC model was established.

#### Results

##### Gaming motives and game genres

**Fig. 1.**
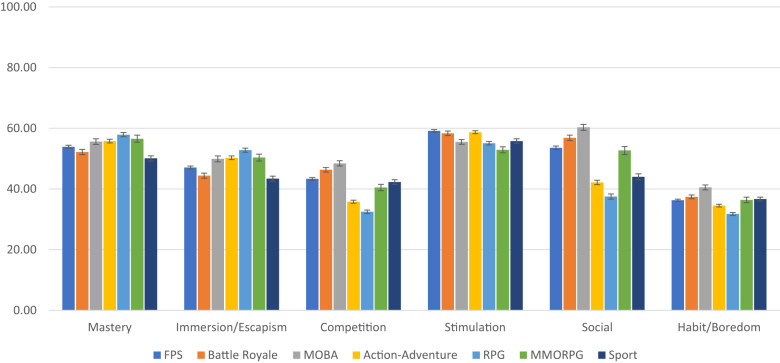
Gaming motivation scores compared across the seven most popular gaming genres in the study sample *Note*. 95% confidence intervals are presented on the bar charts. FPS = first-person shooter; MOBA = multiplayer online battle arena; RPG = role-playing game; MMORPG = massively multiplayer online role-playing game. FPS group (*n* = 5,307); Battle Royale group (*n* = 1,835); MOBA group (*n* = 1,434); Action-adventure group (*n* = 3,463); RPG group (*n* = 2,456); MMORPG group (*n* = 978); Sport group (*n* = 1,739). The summarized sample size of the seven groups exceeds the total sample size because groups partially overlap

To test the associations between gaming motives and game genres, we first computed a correlation matrix (Supplemental Table S7 in the online supplemental materials). Correlations were very weak in general, ranging from −0.251 to 0.201, the former being between the Competition higher-order motive and the RPG genre. The highest positive correlation coefficient found was 0.201 between the Social higher-order motive and the MOBA genre. Second, we created a graphical illustration of the motives and the seven most popular genres in our sample (Fig. 1). Gaming genre variables were dichotomized (respondents who indicated a percentage equal to or higher than 30% in a particular genre were considered players with a preference for that genre and were included in the analysis).

According to the results, several significant differences in all six motives were found between the players of the seven gaming genres, but the magnitude of these differences was generally small. The largest differences were found in the Social and Competition higher-order motive scores between the players with a preference for the MOBA and RPG genres (16-point difference in the Competition higher-order motive scores and 23-point difference in the Social higher-order motive scores on a 100-point scale). Furthermore, players with a preference for the MOBA genre had the highest scores in the Social, Competition, and Habit/Boredom higher-order motives; players with a preference for the RPG genre had the highest scores in the Mastery and Immersion/Escapism higher-order motives; and players with a preference for action-adventure, first-person shooter, and battle royale genres had the highest scores in the Stimulation higher-order motive.

##### MIMIC model

A MIMIC analysis was done to test the associations between the six higher-order motivational factors and numerous predictors. According to the results, associations between relevant personality and psychological variables and the six higher-order motives were as expected (see Table 4 for the standardized regression coefficients of the MIMIC model and Supplemental Table S8 in the online supplemental materials for the correlation coefficients of the variables included in the model). For instance, the Mastery higher-order motive was moderately associated with positive affect and weakly with sensation seeking. The Immersion/Escapism higher-order motive was positively and moderately associated with negative affect and weakly with perceived stress, and it was negatively associated with competitiveness with weak effect size. The Competition higher-order motive was most strongly associated with competitiveness, and it was also weakly associated with negative affect and negatively associated with age with weak effect size. The Stimulation higher-order motive was also weakly associated with competitiveness, sensation seeking, and negative affect. The Social higher-order motive was moderately associated with sociability and negatively associated with age with medium effect size, and the Habit/Boredom higher-order motive was moderately associated with negative affect and negatively associated with positive affect and age, having medium effect sizes in both cases.

**Table 4.** How relevant psychological variables predict the six main motivations: path coefficients of multiple indicator multiple cause model (*N* = 14,740)

Predictor	Gaming motive					
	Mastery	Immersion/Escapism	Competition	Stimulation	Social	Habit/Boredom
Self-esteem	−0.096***	−0.063***	−0.023*	0.052***	−0.087***	−0.036
Positive affect	0.344***	0.123***	0.033**	0.092***	0.097***	−0.220***
Negative affect	0.024	0.242***	0.154***	0.128***	0.010	0.250***
Sociability	−0.087***	−0.103***	−0.016	−0.020	0.261***	0.067**
Competitiveness	0.012	−0.148***	0.604***	0.187***	0.133***	−0.120*
Perceived stress	0.077***	0.188***	0.044***	0.053**	0.030*	0.148***
Sensation seeking	0.123***	0.080***	−0.008	0.174***	0.083***	0.050**
Age	−0.067***	−0.080***	−0.155***	−0.045	−0.250***	−0.266***
Gender	0.056***	0.062***	−0.034***	−0.060*	−0.041***	−0.103***

*Note*. Effect sizes are provided as standardized betas. Gender: males were coded as 0, females as 1.

*P < 0.05. **P < 0.01. ***P < 0.001.

In addition, the relation of COVID-19 distress with the six higher-order motivational factors was analyzed (see Supplemental Figure S2 in the online supplemental materials). Results showed that the relatively small group whose mood has improved due to the pandemic situation reported significantly higher scores across all motives than those whose mood has not changed or worsened. Besides, the group whose mood worsened scored higher on Immersion/Escapism and Habit/Boredom motives and lower on Social motive than the group whose mood has not changed.

### Stage 4: Associations of the higher-order motivational dimensions with depression symptoms, gaming disorder symptoms, and gaming time

Finally, in Stage 4, as the last step of the validation process, we examined how the six higher-order motives mediated between depression symptoms and GD symptoms and gaming time. We aimed to compare the mediation model with similar models reported previously^3^ (e.g., Ballabio et al., 2017; Bányai et al., 2019; Király et al., 2015).

#### Statistical analysis

To test the direct and the indirect effects (via the higher-order gaming motives) of depression symptoms on GD symptoms and gaming time, we performed structural regression analyses within structural equation modelling (SEM) with the MLR estimation method. We assumed that depression symptoms have both a direct and indirect effect (via the mediating effect of the six higher-order gaming motives) on GD symptoms and gaming time. Depression symptoms were measured with the short version of the CES-D and introduced in the model as a continuous observed variable. GD symptoms were assessed by the summarized score of the dichotomized IGDT-10 items, except for Item 8 (i.e., escaping or relieving a negative mood; see Measures section), and implemented in the model as a continuous observed variable. Gaming time was calculated from time spent on games during weekdays and weekend days and was also entered in the model as a continuous observed variable. Higher-order gaming motives were introduced in the model as continuous latent variables as defined in the ESEM analysis. In addition, Pearson's correlation coefficients of the variables included in the mediation model were calculated.

#### Results

**Fig. 2.**
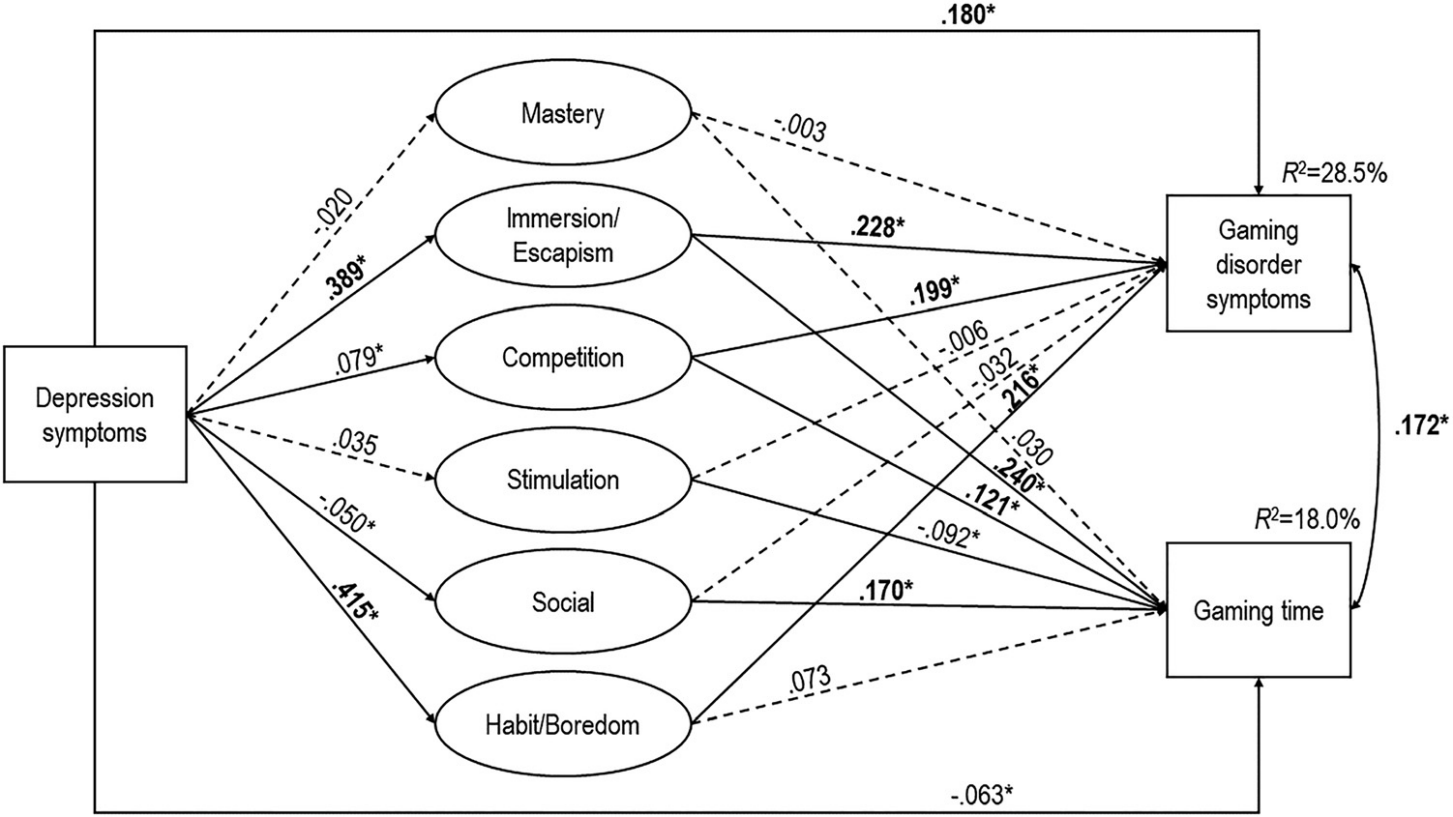
Mediation model between depression symptoms and gaming disorder symptom severity and gaming time (*N* = 14,740) *Note*. Values on single-headed arrows are standardized regression coefficients (*β*). The value on the double-headed arrow represents a correlation coefficient. Due to the large sample size, only *P < 0.001 was considered as a significant effect. Solid lines represent significant standardized regression coefficients. Dashed lines represent non-significant standardized regression coefficients. Bold letters represent considerable standardized regression coefficients (*β* > 0.1). Gaming disorder symptoms were calculated by summarizing the dichotomized Ten-Item Internet Gaming Disorder Test items, except for Item 8 (i.e., escaping or relieving a negative mood). We removed Item 8 because its content conceptually overlapped with the higher-order motive Immersion/Escapism, which could have biased the results. The model was controlled for age and gender. However, to ease the interpretation of the figure, correlation coefficients between the latent variables and the covariate effects of gender and age are not shown (see Supplemental Table S10 in the online supplemental materials)

According to the results of the mediation model (see Fig. 2 for the model, Table 5 for the mediation pathways, Supplemental Table S9 in the online supplemental materials for the predictive effects in the model, and Supplemental Table S10 in the online supplemental materials for the correlation coefficients between the variables included in the model), depression symptoms had a significant direct effect on GD symptoms (*β* = 0.180, *P* < 0.001), as well as on four of six higher-order gaming motives, the strongest effects being on Habit/Boredom (*β* = 0.415, *P* < 0.001) and Immersion/Escapism (*β* = 0.389, *P* < 0.001). Regarding the associations between motives and GD symptoms, Immersion/Escapism, Habit/Boredom, and Competition motives had considerable effect sizes (*β* = 0.228, *β* = 0.216, and *β* = 0.199, respectively).

**Table 5.** Mediation pathways between depression symptoms and gaming disorder (gd) symptoms and gaming time (*N* = 14,747)

	GD symptom severity	Gaming time
Total effect	0.375***	0.058***
Total direct effect	0.180***	−0.063***
Total indirect effect	0.195***	0.121***
via Mastery	0.000	−0.001
via Immersion/Escapism	0.089***	0.093***
via Competition	0.016***	0.010***
via Stimulation	0.000	−0.003*
via Social	0.002	−0.009***
via Habit/Boredom	0.089***	0.030*

*Note*. Effect sizes are provided as standardized betas. GD symptoms were calculated by summarizing the dichotomized Ten-Item Internet Gaming Disorder Test items, except for Item 8 (i.e., escaping or relieving a negative mood). We removed Item 8 because its content conceptually overlapped with the higher-order motive Immersion/Escapism, which could have biased the results.

*P < 0.05. ***P < 0.001.

In relation to the indirect effect between depression symptoms and GD symptoms, three paths were statistically significant at the *P* < 0.001 level: (a) depression symptoms → Immersion/Escapism → GD symptoms (*β* = 0.089, *P* < 0.001); (b) depression symptoms → Habit/Boredom → GD symptoms (*β* = 0.089, *P* < 0.001); and (c) depression symptoms → Competition → GD symptoms (*β* = 0.016, *P* < 0.001). However, the latter pathway had a negligible effect size. The mediation pathways added up to a total standardized indirect effect size of 0.195 (*P* < 0.001). The proportion of the mediated effect in the total effect was 52%. Therefore, higher levels of depression symptoms were associated with higher Immersion/Escapism, Habit/Boredom, and Competition motives that were associated with higher GD symptoms. The full model explained 28.5% of the total variance of GD symptoms.

Furthermore, depression symptoms had a negative direct effect on gaming time, with a very small effect size (*β* = −0.063, *P* < 0.001). Regarding the association between motives and gaming time, Immersion/Escapism, Social, and Competition had considerable effect sizes (*β* = 0.240, *β* = 0.170, and *β* = 0.121, respectively). Comparing the correlation and path coefficient between Stimulation and gaming time (*r* = 0.133, *P* < 0.001 vs. *β* = −0.092, *P* < 0.001), we suspect a negative suppressor effect (Ludlow & Klein, 2014); therefore, we have not interpreted that association.

In relation to the indirect effect between depression symptoms and gaming time, only the pathway through Immersion/Escapism was close to 0.1 (*β* = 0.093, *P* < 0.001). The path through Habit/Boredom had an effect size of *β* = 0.030, *P* = 0.021, whereas the one through Competition was *β* = 0.010, *P* < 0.001 and the one through Social motive was *β* = −0.009, *P* < 0.001. The mediation pathways added up to a total standardized indirect effect size of 0.121 (*P* < 0.001). Consequently, higher levels of depression symptoms were associated with a stronger Immersion/Escapism motive that was associated with higher GD symptoms. The full model explained 18.0% of the total variance of gaming time. Finally, the association between gaming time and GD symptoms was 0.172 (*P* < 0.001) in the model.

## Discussion

In this study, we aimed to create a comprehensive and genre-neutral (applicable to all types of video games) gaming motivation inventory (the GMI) and test its psychometric properties and associations with personality and psychological constructs. After developing a large item pool identified by a systematic literature review, we retained 26 motives that clustered into six higher-order motivational dimensions. The findings support the validity and good psychometric properties of the basic factors, as well as the higher-order structure.

The six higher-order motives retained in the GMI appear to be sufficiently comprehensive to cover those in most previous video gaming motivational models. For example, the higher-order motives in our model cover the three overarching motivational components (Achievement, Social, and Immersion) from Yee's research (2006) to a high degree. The Achievement component is covered by two of the six higher-order motives retained in the GMI: Mastery and Competition. In our model, these motives were clearly separated; the first refers to advancing in games, exploring, mastering game mechanics, and completing tasks and challenges, whereas the second refers to competition and winning, as well as social comparison and recognition within the gaming community. Yee's higher-order Social component is covered by the higher-order motive of the same name in the GMI. Finally, Yee's Immersion higher-order component is mostly covered by the Immersion/Escapism higher-order factor in the GMI, and some of the subcomponents of Immersion (discovery and customization) can be found in the GMI's Mastery higher-order motive. Although the motives escape, fantasy, and coping appear as distinct motives in the MOGQ (Demetrovics et al., 2011; Wu, Lai, Yu, Lau, & Lei, 2016), they belong to one higher-order motive in the GMI: Immersion/Escapism. The reason for this is most probably the considerable correlation between these motives in both studies, caused by their potentially overlapping content. Furthermore, in a study on Turkish students and video game players, Evren, Evren, Dalbudak, Topçu, and Kutlu (2020) conducted an EFA on the MOGQ items, which resulted in a six-factor structure, in which coping and escape items loaded on the same factor.

Another interesting point when comparing these motivational models is that competition and community cluster together in Quantic Foundry's model in the overarching dimension called Social, and Yee argues that gamers who enjoy social interaction tend to enjoy other types of social interaction too, including competing with fellow players (Yee, 2017). In line with this, the Competition and Social higher-order motives have the highest correlation (*r* = 0.48) in the GMI.

To test the construct validity of the six higher-order motivational dimensions in our model, we first checked their associations with gaming genres, relevant personality and psychological variables, age, and gender. The associations between the six higher-order motives and game genres were generally weak. The reason for this may be that video games today are designed in a way to motivate gamers in several different and complementary ways; consequently, there are few differential links between distinct game genres and specific motives. MMORPGs, for instance, are known for the large variety of different gameplay choices that they offer. They can be highly competitive through the so-called player versus player challenges and highly cooperative through the so-called player versus environment challenges, but they also offer quests for players who like to play alone and focus more on exploration, customization, or role playing an imaginary character (Yee, 2006b). Furthermore, shooter games, battle royale games, and the MOBA genre have both strong competition and social elements through team-based matches. To succeed as a team member, one must be competitive, strategic, and cooperative at the same time.

The associations between the higher-order gaming motives and relevant personality and psychological variables (i.e., self-esteem, positive and negative affect, perceived stress, sensation seeking, sociability, competitiveness) were in line with expectations and previous findings supporting the validity of the motivational structure. The Competition higher-order motive was strongly associated with competitiveness and the higher-order motive Social was moderately associated with sociability. Stimulation was weakly associated with competitiveness, sensation seeking, and negative affect. Stimulation is about playing for the excitement and enjoying action and destruction, and it is associated with game genres that are highly competitive and full of fast-paced action such as first-person shooters. The Mastery higher-order motive had the highest association with positive affect. This indicates that playing to complete challenges, master skills, and explore different options is associated with a positive affective state, which is in line with findings from previous studies that have applied the self-determination theory to video games and report that perceived in-game competence and autonomy are associated with game enjoyment and increased well-being (Ryan et al., 2006).

Immersion/Escapism was moderately associated with negative affect and weakly with perceived stress and positive affect. This aligns well with previous studies that consistently report a moderate association between psychiatric symptoms and escapism, fantasy, and coping motives (i.e., playing games to reduce stress; Ballabio et al., 2017; Bányai et al., 2019; Király et al., 2015). This association suggests that players with different psychological problems such as depression or anxiety symptoms are more likely to immerse themselves in video games to avoid facing everyday difficulties, try to reduce stress, or get into a better mood. However, the “only” moderate effect size of these associations, as well as the weak association between this motive and positive affect, indicate that Immersion/Escapism is not always or inherently a maladaptive gaming motivation, because players may also play to escape without experiencing adverse consequences (Giardina, Starcevic, et al., 2021; Kardefelt-Winther, 2014b).

Habit/Boredom was positively associated with negative affect and negatively with positive affect with moderate effect sizes; it was also weakly associated with perceived stress, which indicates the maladaptive nature of this motive. This is in line with previous findings. For instance, Peracchia, Presaghi, and Curcio (2019) reported a moderate association between amotivation (i.e., one of the important motives of the Habit/Boredom higher-order motive) and anxiety and depression symptoms. Furthermore, Mills, Milyavskaya, Heath, and Derevensky (2018) found that amotivation was moderately related to needs frustration in daily life, namely competence, autonomy, and relatedness frustration. Similarly, Lafrenière et al. (2012) found that amotivation was weakly and negatively associated with perceived in-game autonomy, competence, and relatedness. These findings suggest that those individuals who play games without being motivated, or keep playing despite not perceiving any good reasons for it, are more likely to also experience psychiatric problems, stress, and needs frustration both in their lives and in games; relatedly, they are more likely to be in a negative affective state. Finally, self-esteem had negligible effect sizes on all six higher-order gaming motives, suggesting that one's subjective evaluation of own worth is not related to the reasons for play.

Age had a considerable (moderate or weak) negative association with Habit/Boredom, Social, and Competition higher-order motives. The latter two are in line with the results of other studies that reported that younger players are more competitive and more social than older players (Demetrovics et al., 2011; Yee, 2016) and that competition and social motives are strongly related (Yee, 2015b). Furthermore, it is plausible that younger players are more prone to play to avoid boredom, as they have fewer responsibilities and more free time than older players do. Gender differences in the six higher-order motives were relatively small. Female players scored higher in the Mastery and Immersion/Escapism higher-order motives. The latter is in line with previous studies (Király et al., 2015), whereas results regarding the former are controversial. In his early work, Yee (2006) found that male players had higher scores on advancement and mechanics motives, which have similar content to the Mastery higher-order motive in the GMI. In later work (Yee, 2015a), design, completion, and discovery motives were more peculiar to females, whereas challenge and strategy motives were higher for males. These motives all appear in our higher-order motive labeled Mastery. Male players scored higher in the Competition, Stimulation, Social, and Habit/Boredom higher-order motives. These results are mostly in line with previous studies but not entirely. The competition motive was generally stronger in male players (Yee, 2006a, 2015a; Demetrovics et al., 2011), as was the stimulation motive (Yee, 2015a). The social motive was found to be stronger for females in the study of Demetrovics et al. (2011) and Yee (2006), whereas the community motive was higher for males in the Quantic Foundry model. The boredom motive was assessed only among augmented reality game players (Zsila et al., 2017), where no gender differences were found. However, boredom proneness is generally higher in males than in females (Sundberg, Latkin, Farmer, & Saoud, 1991), which is consistent with the result that males are more motivated to play to avoid boredom or because gaming becomes a habitual activity for them.

According to the literature, certain gaming motives (i.e., escape, competition, fantasy) mediate between psychiatric distress and problematic gaming (Ballabio et al., 2017; Bányai et al., 2019; Király et al., 2015). To further test the construct validity of the six higher-order motivational dimensions, we tested a similar model with depression symptoms as the predictor variable, the six higher-order motives as mediators, and GD symptoms and gaming time as outcome variables. We included gaming time as a second outcome variable to see how motives relate to it compared with GD.

Regarding the first outcome variable, depression symptoms had a significant direct effect on GD symptoms and an even stronger indirect effect via Immersion/Escapism, Habit/Boredom, and Competition higher-order motives, the latter having a much weaker effect size than the others. These results are in line with previous findings and suggest that depression symptoms lead to an apathetic, escapist type of play by reducing positive affect and goal-seeking behavior (Nutt et al., 2007). The individual thinks there is no point to anything, including gaming, but keeps playing to avoid boredom and negative emotions caused by real-life problems, which appears to work as a maladaptive coping mechanism and, in certain cases, may lead to negative (addiction-like) real-life consequences. However, the proposed direction of the model (i.e., depression symptoms strengthening escapism and boredom motives and causing GD) requires further studies with a longitudinal design.

The Immersion/Escapism motivation can be considered a form of distraction, an emotion regulation strategy when the person diverts his or her attention away from an emotionally difficult situation (Sheppes & Gross, 2012). When an individual is confronted with high-intensity negative emotions, distraction can serve as an effective strategy—requiring minimal effort—to block the information related to the negative emotions by diverting attention to neutral stimuli unrelated to the original emotions (Campbel-Sills & Barlow, 2007). Unfortunately, this strategy is expected to be ineffective in the long run (Kross & Ayduk, 2008) because it hinders elaborated processing of the aversive emotional event (Campbel-Sills & Barlow, 2007; Sheppes & Gross, 2012). The individual may feel better while playing; however, the negative emotions persist and reappear as soon as the person stops playing. If the person has a rich variety of different emotion regulation strategies that can be flexibly implemented depending on the situation (Aldao, Sheppes, & Gross, 2015), GD is unlikely to develop. However, if this is the main strategy that the person uses to ease feelings of distress, the risk of problematic gaming increases.

The Habit/Boredom higher-order motive includes amotivation and boredom, which were both found to be associated with problematic gaming (Mills et al., 2018; Zsila et al., 2017). It is plausible to assume that the individual whose main or only reason to play games is to decrease negative affective states such as boredom lacks the large palette of emotion regulation strategies and flexibility to adapt to different situations. However, this proposal needs further examination.

The weakest mediator was the Competition higher-order motive. In this case, a possible explanation is that players who experience depression symptoms also lack sources of success in their lives and may be more prone to play to achieve success in video games and a respected status within the gaming community. Although there is no problem with playing games to attain success, if this becomes the main or only source of success for the individual, he or she may start playing in a compulsive manner, which increases the risk of GD.

Regarding the second outcome variable, depression symptoms had a very small negative direct effect on gaming time, but had a considerable indirect effect via the Immersion/Escapism, Habit/Boredom, and Competition higher-order motives, the latter two having much weaker effect sizes than the first. Interestingly, the direct effects between Immersion/Escapism and GD and between Immersion/Escapism and gaming time were of similar size in this study, whereas the former was much stronger in a previous study (Király, Tóth, Urbán, Demetrovics, & Maraz, 2017). It appears that Immersion/Escapism is a motive that is associated with a relatively high gaming time, followed by the Social and Competition higher-order motives, which is reasonable given that in-game activities related to these motives are highly time-consuming. For instance, immersing oneself in a character's story and escaping everyday problems via gaming, socializing, and competing with other players all need substantial amounts of time. The association of the Habit/Boredom higher-order motive and gaming time was weak, suggesting that this motive is related to less time-consuming gaming activities. Finally, the weak correlation between gaming time and GD symptoms supports previous findings, which suggest that gaming time alone is not a good predictor of gaming problems; in other words, intense video gaming in the majority of cases is not problematic (Griffiths, 2010; Király, Tóth, et al., 2017).

### Strengths and limitations

Strengths of the study comprise the systematic literature review which was used to create the initial item pool and which contributed to the development of a truly comprehensive measure. A series of statistical analyses and numerous variables were used to validate the instrument, which shows good psychometric properties. The motivation inventory can both be used in research and in the clinical practice (see the implications in the next section).

Nevertheless, the study also has several limitations. The sample was a self-selected convenience sample; therefore, it is not representative of the entire gamer population. However, research demonstrates (Khazaal et al., 2014) that online gaming surveys primarily attract those individuals who are more involved in games, and therefore self-selected gamer samples are particularly suitable for our research aims. Moreover, the study sample was large, and the majority of respondents were highly engaged gamers who spent 28 h per week on average playing video games, mostly first-person shooter, open-world action-adventure, RPGs, and battle royale games. Self-report assessment has its inherent limitations, such as memory recall bias and social desirability bias. The cross-sectional study design is suitable for scale development, but it is not suitable to explore causal or temporal associations between the motives and external variables. Another limitation is the length of the questionnaire; the list of motives comprised 100 items, which may have caused fatigue in some of the respondents and led to a considerable attrition rate. Furthermore, the operationalization of items for two motives (graphics and story) was not ideal, as they were too positively phrased, which is also mirrored by the high negative skewness values of these motives. These items should be asked differently in future studies; for instance, respondents should be asked to rate the importance of these features when they play instead of whether they prefer good graphics and stories.

### Practical use of the inventory, implications, and future research directions

The GMI can be used in two ways. First, it can serve as a profiling tool, useful in both research and clinical settings. Scores on the 26 motives provide a motivational profile of the individual, comprising information regarding the latent psychological needs motivating him or her in gaming, and may also provide information regarding everyday motives and behaviors. Second, given the length of the inventory (88 items), parts of it can be used alone. If, in research or clinical settings, there is a specific interest in certain motives and underlying psychological needs, subscales or specific motivation factors can be used alone to explore them. The use of GMI subscales is supported by adequate internal reliability and high factor loadings of the items (all Cronbach's alpha >0.70, the majority of factor loadings between 0.6 and 0.9, and only a few above 0.5; see Supplemental Table S4 in the online supplemental materials). It remains the task of future research to develop a shorter scale covering the six higher-order motives.

The comprehensive and genre-neutral motivational item pool, and the findings regarding the 26 basic motives and the six higher-order motives, is an important contribution to the video gaming research field. Given that gaming is one of the most popular leisure time activities, it is crucial to understand why people of different genders and ages are pulled toward video games and what their main motives are to pursue this activity as a prominent hobby. Furthermore, there are important clinical implications. Research consistently shows that motivations play an important role in the development and maintenance of addictive behaviors, and this study suggests that Immersion/Escapism and Habit/Boredom motives constitute a risk factor for GD symptoms. Interventions should always take gaming motives into account and use them to explore and address underlying psychological mechanisms that lead to pathological behavior. However, it remains the task of future research to identify and more deeply examine the psychological processes underlying gaming motives, as well as to explore their temporal stability and predictive power for GD symptoms in large-scale longitudinal studies and across cultures.

## Funding sources

ZD's contribution was supported by the Hungarian National Research, Development and Innovation Office (KKP126835; K128614; K134807). OK was supported by the János Bolyai Research Scholarship of the Hungarian Academy of Sciences and by the ÚNKP-21-5 New National Excellence Program of the Ministry for Innovation and Technology from the source of the National Research, Development and Innovation Fund.

## Authors' contribution

OK, UR, JB, DLK, and ZD took part in conceptualization. OK and UR conducted the formal analysis. OK, UR and ZD designed the methodology. ZD and OK were responsible for funding acquisition. OK, EP and PK took part in the investigation. OK and PK was responsible for project administration. OK, JB, DLK wrote the original draft. All authors took part in the review and editing process.

## Conflict of interest

ELTE Eötvös Loránd University receives funding from the Szerencsejáték Ltd. to maintain a telephone helpline service for problematic gambling. ZD has also been involved in research on responsible gambling funded by Szerencsejáték Ltd. and the Gambling Supervision Board and provided educational materials for the Szerencsejáték Ltd's responsible gambling program. The University of Gibraltar receives funding from the Gibraltar Gambling Care Foundation. However, these funding aren't related to this study and the funding institution had no role or any influence on this publication. ZD has been member of a WHO advisory group on the public health consequences of addictive behaviors. In this capacity he has been eligible for travel support from WHO or the host center to attend advisory group meetings but have not been remunerated for their work. ZD is the Editor-in-Chief of the Journal of Behavioral Addictions. JB, DLK and UR are Associate Editors of the Journal of Behavioral Addictions.

The other authors do not have conflicts of interest.

## Open science framework

All data and analysis code are available at the open science framework (OSF): https://osf.io/tfhjx/.

## Supplementary material

## Footnotes

1 Three popular-science articles in Hungarian appeared that described part of the findings of the present research project: https://www.gamestar.hu/extralife/mi-motivalja-a-magyar-gamert-287693.html; https://www.gamestar.hu/extralife/sokat-mesel-el-a-szemelyisegedrol-ahogy-jatszol-289916.html; https://www.gamestar.hu/extralife/hogyan-vehetjuk-eszre-a-jatekfuggoseg-jeleit-293161.html.

2 Qualtrics software provides the option to randomize the items of a scale for each respondent individually returning the items in the original order when data is downloaded.

3 Mediation models reported previously used general psychiatric distress (an index composed of multiple psychiatric symptoms including depression and anxiety symptoms) as a predictor. In this study, we decided to only assess depression symptoms as one of the most common psychiatric symptoms, and because of its high correlations with other psychiatric symptoms such as anxiety symptoms (Jacobson & Newman, 2017).

## Appendix A

### Gaming Motivation Inventory (GMI) – structured version

Instruction: People play video games for different reasons. You can see such reasons listed below. Please indicate how much each statement corresponds in your case. Response options range from 1 (“It does not correspond at all”) to 7 (“It corresponds exactly”). There are no right or wrong answers. We are curious to find out why you play.

**Table:**

Factor	Item							
**Why do you play video games? I play video games…**								
Advancement	1. because I like the feeling of continuous advancement	1	2	3	4	5	6	7
	2. because I like to advance in games	1	2	3	4	5	6	7
	3. because I like it when I get to the next level/stage/point in games	1	2	3	4	5	6	7
Amotivation	4. I used to have good reasons, but now I am asking myself if I should continue	1	2	3	4	5	6	7
	5. Honestly, I don't know; I have the impression that I'm wasting my time	1	2	3	4	5	6	7
	6. It is not clear anymore; I sometimes ask myself if it is good for me	1	2	3	4	5	6	7
Autonomy	7. because I can determine for myself what I do in games	1	2	3	4	5	6	7
	8. because I can play the games according to my preferences	1	2	3	4	5	6	7
	9. because they provide me with interesting options and choices	1	2	3	4	5	6	7
	10. because I experience a lot of freedom in games	1	2	3	4	5	6	7
Boredom	11. because I am bored	1	2	3	4	5	6	7
	12. to pass the time	1	2	3	4	5	6	7
	13. because there is nothing else to do	1	2	3	4	5	6	7
Competence	14. because when I perform well, it makes me feel good about myself	1	2	3	4	5	6	7
	15. because when I'm successful, it boosts my self-esteem	1	2	3	4	5	6	7
	16. because I feel very capable and effective when playing	1	2	3	4	5	6	7
Competition	17. because I like competing with others	1	2	3	4	5	6	7
	18. because I like to win	1	2	3	4	5	6	7
	19. because I like to be better than others	1	2	3	4	5	6	7
Completion	20. until I get 100% on them, completing everything possible	1	2	3	4	5	6	7
	21. until I unlock all achievements	1	2	3	4	5	6	7
	22. until I master all elements of a game	1	2	3	4	5	6	7
Coping	23. because it helps me get my anger out	1	2	3	4	5	6	7
	24. because it helps me get rid of stress	1	2	3	4	5	6	7
	25. because it helps me get into a better mood	1	2	3	4	5	6	7
Escape	26. to avoid thinking about some of my real-life problems or worries	1	2	3	4	5	6	7
	27. because gaming helps me to forget about daily hassles	1	2	3	4	5	6	7
	28. to forget about unpleasant things or offences	1	2	3	4	5	6	7
	29. because gaming helps me escape reality	1	2	3	4	5	6	7
Exploration + Mechanics	30. because I like to explore different elements or possibilities of the game	1	2	3	4	5	6	7
	31. because I like to experiment with different ways to play the game	1	2	3	4	5	6	7
	32. because I like to figure out how specific game elements work in detail	1	2	3	4	5	6	7
	33. because I like to discover/learn the game mechanics thoroughly	1	2	3	4	5	6	7
Fantasy	34. because I can do things that I am unable to do or I am not allowed to do in real life	1	2	3	4	5	6	7
	35. because I can be in another world	1	2	3	4	5	6	7
	36. to be somebody else or somewhere else for a while	1	2	3	4	5	6	7
	37. because I feel immersed in the virtual world	1	2	3	4	5	6	7
Financial	38. because I have the possibility to earn money	1	2	3	4	5	6	7
	39. because I have the chance to earn some extra income	1	2	3	4	5	6	7
	40. because I can make some money	1	2	3	4	5	6	7
Game skills	41. because I like to perform to the best of my ability	1	2	3	4	5	6	7
	42. because I like to improve specific gaming skills	1	2	3	4	5	6	7
	43. because I like to continuously improve my own gameplay	1	2	3	4	5	6	7
	44. because I like to practice and master a game	1	2	3	4	5	6	7
Identity	45. because playing games is a meaningful activity	1	2	3	4	5	6	7
	46. because it is an extension of myself	1	2	3	4	5	6	7
	47. because it is an integral part of my life	1	2	3	4	5	6	7
	48. because it has personal significance to me	1	2	3	4	5	6	7
	49. because this game/gaming is in harmony with the other activities in my life	1	2	3	4	5	6	7
Introjected regulation	50. because I must play to feel good about myself	1	2	3	4	5	6	7
	51. because otherwise I would feel bad about myself	1	2	3	4	5	6	7
	52. because I feel that I must play regularly	1	2	3	4	5	6	7
Recreation	53. because it is fun	1	2	3	4	5	6	7
	54. for recreation	1	2	3	4	5	6	7
	55. to relax	1	2	3	4	5	6	7
Skill development	56. because gaming sharpens my senses	1	2	3	4	5	6	7
	57. because it improves my skills	1	2	3	4	5	6	7
	58. because it improves my concentration	1	2	3	4	5	6	7
	59. because it improves my coordination skills	1	2	3	4	5	6	7
Social	60. because I can get to know new people	1	2	3	4	5	6	7
	61. because I like playing with others	1	2	3	4	5	6	7
	62. because I feel close to other gamers	1	2	3	4	5	6	7
	63. because I find the relationships I form in games meaningful	1	2	3	4	5	6	7
Status	64. for the prestige of being a good player	1	2	3	4	5	6	7
	65. because I gain recognition and esteem from others	1	2	3	4	5	6	7
	66. because others perceive me as skilled	1	2	3	4	5	6	7
**What kind of gameplay do you prefer? I like video games that…**								
Arousal-action	67. raise the level of adrenaline	1	2	3	4	5	6	7
	68. keep the players on the edge of their seats	1	2	3	4	5	6	7
	69. raise the level of excitement	1	2	3	4	5	6	7
	70. are intense and full of action	1	2	3	4	5	6	7
Cooperation	71. allow players to cooperate with others	1	2	3	4	5	6	7
	72. promote working together in a group	1	2	3	4	5	6	7
	73. require players to work together	1	2	3	4	5	6	7
Customization	74. allow players to customize their in-game objects (e.g., avatar/vehicle/stuff…)	1	2	3	4	5	6	7
	75. provide players with a lot of customization options	1	2	3	4	5	6	7
	76. allow players to personalize their objects/stuff/characters so that those can be unique	1	2	3	4	5	6	7
Destruction	77. allow players to make explosions	1	2	3	4	5	6	7
	78. involve destruction	1	2	3	4	5	6	7
	79. allow players to mess things up	1	2	3	4	5	6	7
Graphics	80. are visually breathtaking	1	2	3	4	5	6	7
	81. have outstanding graphics	1	2	3	4	5	6	7
	82. have good graphics, are beautiful	1	2	3	4	5	6	7
Story	83. involve an interesting story	1	2	3	4	5	6	7
	84. involve an elaborate story that stimulates my emotions	1	2	3	4	5	6	7
	85. involve an immersive narrative	1	2	3	4	5	6	7
Strategy	86. require strategic thinking	1	2	3	4	5	6	7
	87. require planning ahead and making strategic decisions	1	2	3	4	5	6	7
	88. require tactical decision making	1	2	3	4	5	6	7

Recommendations for administration:

1. If the entire inventory is used in epidemiological online surveys, we recommend randomizing the items of the two blocks for each participant individually to avoid systematic missing values on the later part of the item pool because of fatigue that may appear due to the lengths of the inventory. More specifically, we recommend randomizing items 1 to 66 of the first format “Why do you play video games? I play video games…” and then items 67 to 88 of the second format “What kind of gameplay do you prefer? I like video games that…” separately.
2. If only specific motivational factors are used, we recommend randomizing the items of the two question formats separately for all respondents centrally or individually.
3. If the inventory is used in the clinical setting, we recommend using the randomized version provided in Appendix B.

## Appendix B

### Gaming Motivation Inventory (GMI) – randomized version

Instruction: People play video games for different reasons. You can see such reasons listed below. Please indicate how much each statement corresponds in your case. Response options range from 1 (“It does not correspond at all”) to 7 (“It corresponds exactly”). There are no right or wrong answers. We are curious to find out why you play.

**Table:**

Item							
**Why do you play video games? I play video games…**							
1. because I like the feeling of continuous advancement	1	2	3	4	5	6	7
2. I used to have good reasons, but now I am asking myself if I should continue	1	2	3	4	5	6	7
3. because I can determine for myself what I do in games	1	2	3	4	5	6	7
4. because I am bored	1	2	3	4	5	6	7
5. because when I perform well, it makes me feel good about myself	1	2	3	4	5	6	7
6. because I like competing with others	1	2	3	4	5	6	7
7. until I get 100% on them, completing everything possible	1	2	3	4	5	6	7
8. because it helps me get my anger out	1	2	3	4	5	6	7
9. to avoid thinking about some of my real-life problems or worries	1	2	3	4	5	6	7
10. because I like to explore different elements or possibilities of the game	1	2	3	4	5	6	7
11. because I can do things that I am unable to do or I am not allowed to do in real life	1	2	3	4	5	6	7
12. because I have the possibility to earn money	1	2	3	4	5	6	7
13. because I like to perform to the best of my ability	1	2	3	4	5	6	7
14. because playing games is a meaningful activity	1	2	3	4	5	6	7
15. because I must play to feel good about myself	1	2	3	4	5	6	7
16. because it is fun	1	2	3	4	5	6	7
17. because gaming sharpens my senses	1	2	3	4	5	6	7
18. because I can get to know new people	1	2	3	4	5	6	7
19. for the prestige of being a good player	1	2	3	4	5	6	7
20. because I like to advance in games	1	2	3	4	5	6	7
21. Honestly, I don't know; I have the impression that I'm wasting my time	1	2	3	4	5	6	7
22. because I can play the games according to my preferences	1	2	3	4	5	6	7
23. to pass the time	1	2	3	4	5	6	7
24. because when I'm successful, it boosts my self-esteem	1	2	3	4	5	6	7
25. because I like to win	1	2	3	4	5	6	7
26. until I unlock all achievements	1	2	3	4	5	6	7
27. because it helps me get rid of stress	1	2	3	4	5	6	7
28. because gaming helps me to forget about daily hassles	1	2	3	4	5	6	7
29. because I like to experiment with different ways to play the game	1	2	3	4	5	6	7
30. because I can be in another world	1	2	3	4	5	6	7
31. because I have the chance to earn some extra income	1	2	3	4	5	6	7
32. because I like to improve specific gaming skills	1	2	3	4	5	6	7
33. because it is an extension of myself	1	2	3	4	5	6	7
34. because otherwise I would feel bad about myself	1	2	3	4	5	6	7
35. for recreation	1	2	3	4	5	6	7
36. because it improves my skills	1	2	3	4	5	6	7
37. because I like playing with others	1	2	3	4	5	6	7
38. because I gain recognition and esteem from others	1	2	3	4	5	6	7
39. because I like it when I get to the next level/stage/point in games	1	2	3	4	5	6	7
40. It is not clear anymore; I sometimes ask myself if it is good for me	1	2	3	4	5	6	7
41. because they provide me with interesting options and choices	1	2	3	4	5	6	7
42. because there is nothing else to do	1	2	3	4	5	6	7
43. because I feel very capable and effective when playing	1	2	3	4	5	6	7
44. because I like to be better than others	1	2	3	4	5	6	7
45. until I master all elements of a game	1	2	3	4	5	6	7
46. because it helps me get into a better mood	1	2	3	4	5	6	7
47. to forget about unpleasant things or offences	1	2	3	4	5	6	7
48. because I like to figure out how specific game elements work in detail	1	2	3	4	5	6	7
49. to be somebody else or somewhere else for a while	1	2	3	4	5	6	7
50. because I can make some money	1	2	3	4	5	6	7
51. because I like to continuously improve my own gameplay	1	2	3	4	5	6	7
52. because it is an integral part of my life	1	2	3	4	5	6	7
53. because I feel that I must play regularly	1	2	3	4	5	6	7
54. to relax	1	2	3	4	5	6	7
55. because it improves my concentration	1	2	3	4	5	6	7
56. because I feel close to other gamers	1	2	3	4	5	6	7
57. because others perceive me as skilled	1	2	3	4	5	6	7
58. because I experience a lot of freedom in games	1	2	3	4	5	6	7
59. because gaming helps me escape reality	1	2	3	4	5	6	7
60. because I like to discover/learn the game mechanics thoroughly	1	2	3	4	5	6	7
61. because I feel immersed in the virtual world	1	2	3	4	5	6	7
62. because I like to practice and master a game	1	2	3	4	5	6	7
63. because it has personal significance to me	1	2	3	4	5	6	7
64. because it improves my coordination skills	1	2	3	4	5	6	7
65. because I find the relationships I form in games meaningful	1	2	3	4	5	6	7
66. because this game/gaming is in harmony with the other activities in my life	1	2	3	4	5	6	7
**What kind of gameplay do you prefer? I like video games that…**							
67. raise the level of adrenaline	1	2	3	4	5	6	7
68. allow players to cooperate with others	1	2	3	4	5	6	7
69. allow players to customize their in-game objects (e.g., avatar/vehicle/stuff…)	1	2	3	4	5	6	7
70. allow players to make explosions	1	2	3	4	5	6	7
71. are visually breathtaking	1	2	3	4	5	6	7
72. involve an interesting story	1	2	3	4	5	6	7
73. require strategic thinking	1	2	3	4	5	6	7
74. keep the players on the edge of their seats	1	2	3	4	5	6	7
75. promote working together in a group	1	2	3	4	5	6	7
76. provide players with a lot of customization options	1	2	3	4	5	6	7
77. involve destruction	1	2	3	4	5	6	7
78. have outstanding graphics	1	2	3	4	5	6	7
79. involve an elaborate story that stimulates my emotions	1	2	3	4	5	6	7
80. require planning ahead and making strategic decisions	1	2	3	4	5	6	7
81. raise the level of excitement	1	2	3	4	5	6	7
82. require players to work together	1	2	3	4	5	6	7
83. allow players to personalize their objects/stuff/characters so that those can be unique	1	2	3	4	5	6	7
84. allow players to mess things up	1	2	3	4	5	6	7
85. have good graphics, are beautiful	1	2	3	4	5	6	7
86. involve an immersive narrative	1	2	3	4	5	6	7
87. require tactical decision making	1	2	3	4	5	6	7
88. are intense and full of action	1	2	3	4	5	6	7

Motivational factors with their belonging items		
Advancement: 1, 20, 39	Amotivation: 2, 21, 40	Autonomy: 3, 22, 41, 58
Boredom: 4, 23, 42	Competence: 5, 24, 43	Competition: 6, 25, 44
Completion: 7, 26, 45	Coping: 8, 27, 46	Escape: 9, 28, 47, 59
Exploration + Mechanics: 10, 29, 48, 60	Fantasy: 11, 30, 49, 61	Financial: 12, 31, 50
Game skills: 13, 32, 51, 62	Identity: 14, 33, 52, 63, 66	Introjected regulation: 15, 34, 53
Recreation: 16, 35, 54	Skill development: 17, 36, 55, 64	Social: 18, 37, 56, 65
Status: 19, 38, 57	Arousal-action: 67, 74, 81, 88	Cooperation: 68, 75, 82
Customization: 69, 76, 83	Destruction: 70, 77, 84	Graphics: 71, 78, 85
Story: 72, 79, 86	Strategy: 73, 80, 87	
