# Supplementary material for: A comprehensive model to understand and assess the motivational background of video game use: The Gaming Motivation Inventory (GMI)
Source: J Behav Addict. 2022 Aug 8;11(3):796–819. doi: 10.1556/2006.2022.00048 (PMC9872527; doi:10.1556/2006.2022.00048)
Supplement: Supplementary file 1 [file jba-11-796-s001.doc]

**Supplemental Figure S1**

*Flowchart of the Systematic Database Search*
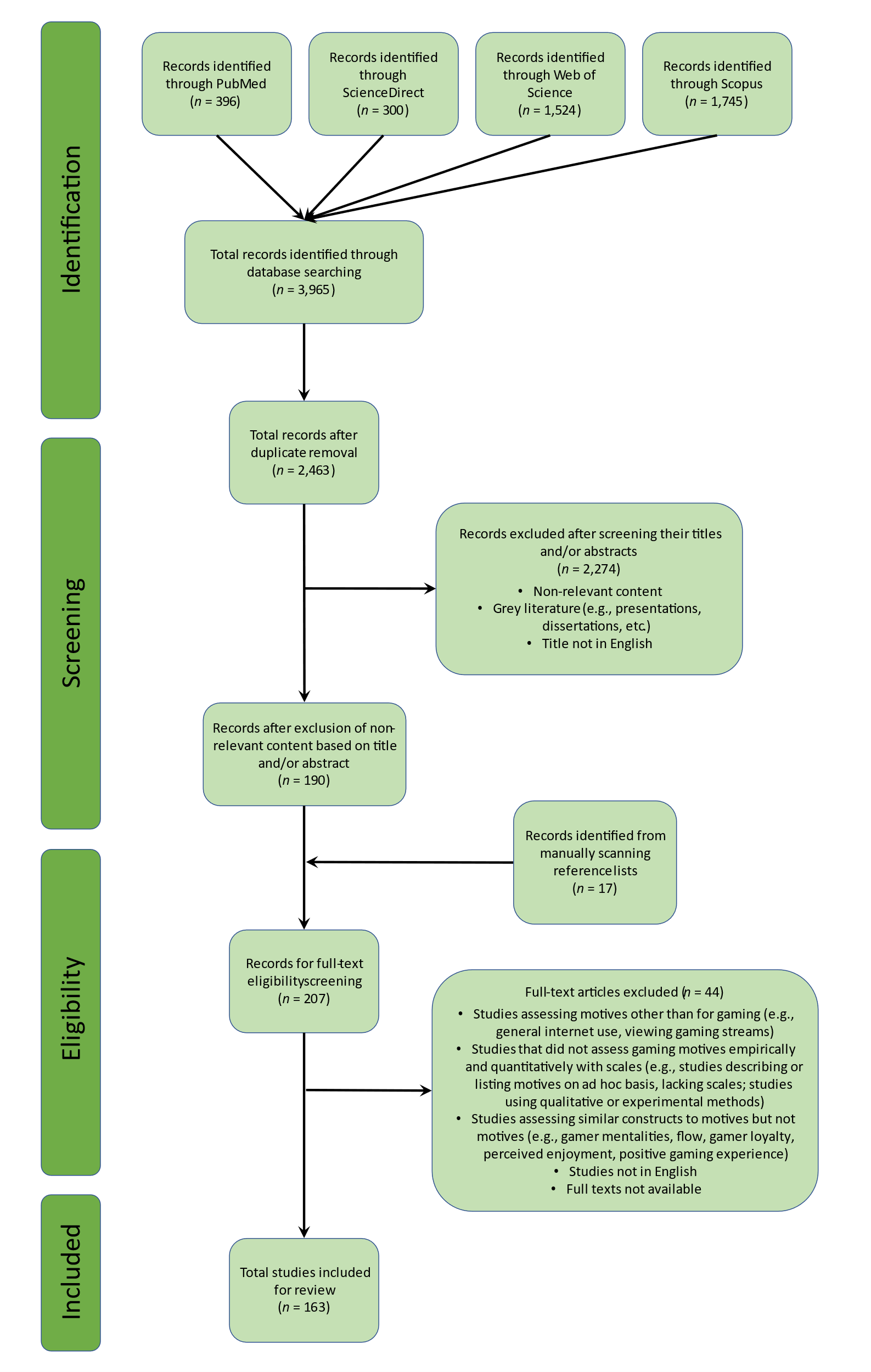


**Supplemental Figure S2**

*Gaming Motivation Scores Compared Across Three COVID-19 Distress Groups (N=12,850)*


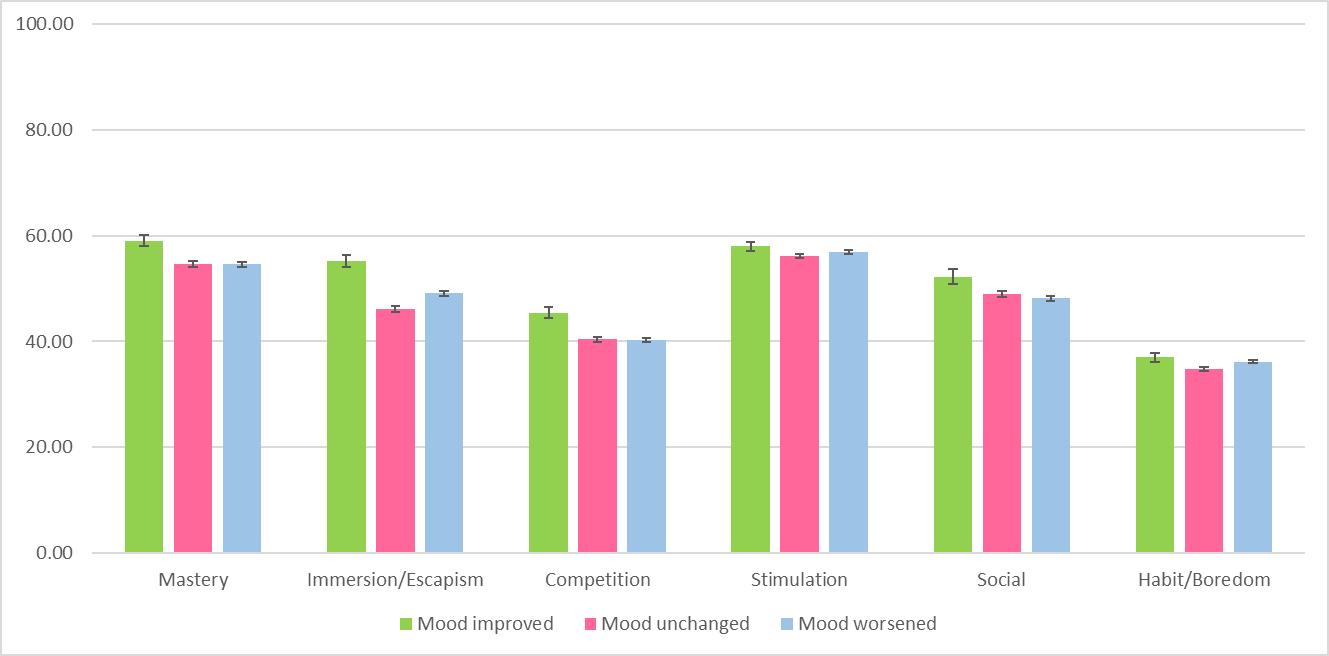


*Note.* 95% confidence intervals are presented on the bar charts. Mood improved, unchanged, or worsened due to the COVID-19 pandemic situation. Mood improved (n=1,063), mood unchanged (n=4,615), mood worsened (n=7,177).

**Supplemental Table S1**

*Most Frequently Used Motivational Instruments F*rom Systematic Literature Review

| Number of occurrences^a^ | Name of the instrument/motivational factor | Source |
| --- | --- | --- |
| 53 | No name | Yee, 2006 |
| 16 | Motives for Online Gaming Questionnaire | Demetrovics et al., 2011 |
| 16 | Passion Scale | Przybylski et al., 2009; Vallerand et al., 2003 |
| 8 | No name | Yee, 2012 |
| 6 | Gaming Motivation Scale | Lafrenière et al., 2012 |
| 6 | Player Experience of Need Satisfaction | Ryan et al., 2006 |
| + Factors from less popular scales that were different or otherwise seemed relevant (e.g., the items had some additional value) | | |
| Validation seeking | | Beard & Wickham, 2016 |
| Agency, pastime, moral self-reaction | | De Grove et al., 2016 |
| Boredom, catharsis | | Ferguson & Olson, 2013 |
| Challenge | | Ghazali et al., 2019 |
| Escapism | | Hagström & Kaldo, 2014 |
| Enjoyment | | Hamari et al., 2019; Wu et al., 2010 |
| Grinding/completion | | Hilgard et al., 2013 |
| Recognition, curiosity | | Hsu et al., 2009 |
| Completionist | | Kahn et al., 2015 |
| Self-presentation | | Li et al., 2015 |
| Arousal | | Sherry et al., 2006 |
| Social | | Wallenius et al., 2009 |
| + Motives from the Gamer Motivation Profile^b^ | | Quantic Foundry: <https://quanticfoundry.com/> |

*Note.* ^a^Number of occurrences in empirical academic papers until November 2019 according to a systematic literature of four databases (PubMed, Science Direct, Web of Science, Scopus) on empirical studies assessing gaming motives.

^b^This scale is not published, but it is the proprietary and copyrighted inventory of Quantic Foundry, a market research company focused on gamer motivation founded by Nick Yee and Nicolas Ducheneaut in 2015. We have reached out to them, but we were not allowed to copy/adapt/use their inventory. Consequently, we have used only those motives (the labels) that are freely available on their website as an inspiration.

**Supplemental Table S2**

*Motivational Item Pool and Its G*eneration

| Why do you play video games? I play video games… (a 7-point Likert scale 1 = *it does not correspond at all*; 7 = *corresponds exactly*) | | | | | |
| --- | --- | --- | --- | --- | --- |
|  | **Factor** | **Item** | **New/Adapted/Original** | **Original scale** | **Original item** |
| 1 | Advancement | because I like the feeling of continuous advancement | New item | New item (inspired from Yee2006) | - |
| 2 | Advancement | because I like to advance in games | New item | New item (inspired from Yee2006) | - |
| 3 | Advancement | because I like it when I get to the next level/stage/point in games | New item | New item (inspired from Yee2006) | - |
| 4 | Amotivation | I used to have good reasons, but now I am asking myself if I should continue | Original item preserved | GAMS | Same |
| 5 | Amotivation | Honestly, I don’t know; I have the impression that I’m wasting my time | Original item preserved | GAMS | Same |
| 6 | Amotivation | It is not clear anymore; I sometimes ask myself if it is good for me | Original item preserved | GAMS | Same |
| 7 | Agency-autonomy | because I can determine for myself what I do in games | Adapted from… | DeGrove | Can determine for yourself what happens in the game |
| 8 | Agency-autonomy | because I can play the games according to my preferences | Adapted from… | DeGrove | Play the game according to your preferences |
| 9 | Autonomy | because they allow me to live memorable experiences | Adapted from… | Passion | This game (activity) allows me to live memorable experiences |
| 10 | Autonomy | because they provide me with interesting options and choices | Original item preserved | PENS | Same |
| 11 | Autonomy | because I experience a lot of freedom in games | Original item preserved | PENS | Same |
| 12 | Boredom | because I am bored | Adapted from… | DeGrove | Play because you are bored |
| 13 | Boredom | to pass the time | Adapted from… | DeGrove | Play to pass the time |
| 14 | Boredom | because there is nothing else to do | Adapted from… | Ferguson&Olson | There is nothing else to do |
| 15 | Competence | because when I perform well, it makes me feel good about myself | Adapted from… | Beard | My sense of self-worth is tied to my overall performance when gaming |
| 16 | Competence | because when I'm successful, it boosts my self-esteem | Adapted from… | Beard | My self-esteem would decrease if I stopped playing the game |
| 17 | Competence | because I feel competent when I play | Adapted from… | PENS | I feel competent at the game |
| 18 | Competence | because I feel very capable and effective when playing | Original item preserved | PENS | Same |
| 19 | Competition | because I like competing with others | Adapted from… | MOGQ | For the pleasure of defeating others |
| 20 | Competition | because competition stimulates me | New item | - | - |
| 21 | Competition-power | because I like to win | Original item preserved | MOGQ | Same |
| 22 | Competition-power | because I like to be better than others | Adapted from… | MOGQ | Because it is good to feel that I am better than others |
| 23 | Competition-power | because I like to be the best | New item | New item (Inspired from QF - Power factor) | - |
| 24 | Completion | until I get 100% on them, completing everything possible | Adapted from… | Hilgard | I’ll play a game until I get a 100% on it, completing everything in the game |
| 25 | Completion | until I unlock all achievements | Adapted from… | Hilgard | I’m excited to unlock achievements or earn trophies in games |
| 26 | Completion | until I master all elements of a game | Adapted from… | Trojan | Like to master all elements of a game |
| 27 | Coping | because it helps me get my anger out | Adapted from… | Ferguson&Olson | It helps me get my anger out |
| 28 | Coping | because it helps me feel less lonely | Adapted from… | Ferguson&Olson | It helps me feel less lonely |
| 29 | Coping | because it helps me get rid of stress | Original item preserved | MOGQ | Same |
| 30 | Coping | because it helps me get into a better mood | Original item preserved | MOGQ | Same |
| 31 | Escape | to avoid thinking about some of your real-life problems or worries | Adapted from… | Hagström | How often do you play so you can avoid thinking about some of your real-life problems or worries? |
| 32 | Escape | because gaming helps me to forget about daily hassles | Original item preserved | MOGQ | Same |
| 33 | Escape | to forget about unpleasant things or offences | Original item preserved | MOGQ | Same |
| 34 | Escape | because it makes me forget real life | Original item preserved | MOGQ | Same |
| 35 | Escape | because gaming helps me escape reality | Original item preserved | MOGQ | Same |
| 36 | Exploration | because of their ability to surprise me in unexpected ways | Adapted from… | Hsu | Playing this game always surprises me in a good way |
| 37 | Exploration | because I like to explore different elements or possibilities of the game | New item | New item (inspired from Yee2006 & QF) | - |
| 38 | Exploration | because I like to discover unconventional ways to play the game | New item | New item (inspired from Yee2006 & QF) | - |
| 39 | Exploration | because I like to experiment with different ways to play the game | New item | New item (inspired from Yee2006 & QF) | - |
| 40 | Fantasy | because I can do things that I am unable to do or I am not allowed to do in real life | Original item preserved | MOGQ | Same |
| 41 | Fantasy | because I can be in another world | Original item preserved | MOGQ | Same |
| 42 | Fantasy | to be somebody else or somewhere else for a while | Adapted from… | MOGQ | To be somebody else for a while |
| 43 | Fantasy | because I feel immersed in the virtual world | Adapted from… | YEE2012 | Feeling immersed in the world |
| 44 | Fantasy/role-play | because I like to role play | Adapted from… | YEE2006 | How often do you role play your character? |
| 45 | Financial | because I have the possibility to earn money | New item | - | - |
| 46 | Financial | because I have the chance to earn some extra income | New item | - | - |
| 47 | Financial | because I can make some money | New item | - | - |
| 48 | Game skills | because I like to perform to the best of my ability | Adapted from… | Ghazali | Playing PG challenges me to perform to the best of my ability |
| 49 | Game skills | because I like to improve specific gaming skills | Adapted from… | MOGQ | Because it improves my skills |
| 50 | Game skills | because I like to continuously improve my own gameplay | New item | - | - |
| 51 | Game skills | because I like to practice and master a game | New item | - | - |
| 52 | Identity | because playing games is a meaningful activity | Adapted from… | DeGrove | I feel that playing games is a meaningful activity |
| 53 | Identity | because it is an extension of me | Original item preserved | GAMS | Same |
| 54 | Identity | because it is an integral part of my life | Original item preserved | GAMS | Same |
| 55 | Identity | because it has personal significance to me | Original item preserved | GAMS | Same |
| 56 | Identity | because this game/gaming is in harmony with the other activities in my life | Adapted from… | Passion | This game (activity) is in harmony with the other activities in my life |
| 57 | Introjected regulation | because I must play to feel good about myself | Original item preserved | GAMS | Same |
| 58 | Introjected regulation | because otherwise I would feel bad about myself | Original item preserved | GAMS | Same |
| 59 | Introjected regulation | because I feel that I must play regularly | Original item preserved | GAMS | Same |
| 60 | Mechanics | because I like to figure out how specific game elements work in detail | Adapted from… | Trojan | I like to figure out how the game works inside and out |
| 61 | Mechanics | because I like to explore the underlying rules of the game | Adapted from… | YEE2006 | How interested are you in the precise numbers and percentages underlying the game mechanics? |
| 62 | Mechanics | because I like to discover/learn the game mechanics thoroughly | Adapted from… | YEE2006 | How important is it for you to know as much about the game mechanics and rules as possible |
| 63 | Recreation | because it is fun | Adapted from… | Hamari | I play Pokémon Go because it is fun |
| 64 | Recreation | for recreation | Original item preserved | MOGQ | Same |
| 65 | Recreation | to relax | New item | - | - |
| 66 | Recreation | because playing gives me a lot of pleasure | Adapted from… | Wu | Playing the online game gives me a lot of pleasure |
| 67 | Skill-development | because gaming sharpens my senses | Original item preserved | MOGQ | Same |
| 68 | Skill-development | because it improves my skills | Original item preserved | MOGQ | Same |
| 69 | Skill-development | because it improves my concentration | Original item preserved | MOGQ | Same |
| 70 | Skill-development | because it improves my coordination skills | Original item preserved | MOGQ | Same |
| 71 | Social | because I can get to know new people | Original item preserved | MOGQ | Same |
| 72 | Social | because I like playing with others | New item | - | - |
| 73 | Social | because I feel close to other gamers | Adapted from… | PENS | I don’t feel close to other players |
| 74 | Social | because I find the relationships I form in games meaningful | Adapted from… | PENS | I find the relationships I form in this game fulfilling |
| 75 | Social | because games are a uniting interest and common topic to discuss with friends | Adapted from… | Wallenius | Games are a uniting interest and common topic to discuss with friends |
| 76 | Status/external regulation | for the prestige of being a good player | Original item preserved | GAMS | Same |
| 77 | Status/external regulation | because I gain recognition and esteem from others | Adapted from… | Hsu | I can gain recognition and esteem from other players |
| 78 | Status/external regulation | because others perceive me as skilled | Adapted from… | Li | I play the SNG because I want other players in this game to perceive me as skilled |
| What kind of gameplay do you prefer? I like video games that… (a seven-point Likert scale 1 = it does not correspond at all; 7 = it corresponds exactly) | | | | | |
| 79 | Arousal-action | raise the level of adrenaline | Adapted from… | Sherry | I find that playing video games raises my level of adrenaline |
| 80 | Arousal-action | keep the players on the edge of their seats | Adapted from… | Sherry | Video games keep me on the edge of my seat |
| 81 | Arousal-action | raise the level of excitement | Adapted from… | Sherry | I play video games because they excite me |
| 82 | Arousal-action | are intense and full of action | New item | - | - |
| 83 | Cooperation | allow players to cooperate with others | Adapted from… | YEE2006 | How much do you enjoy working with others in a group? |
| 84 | Cooperation | promote working together in a group | New item | New item (inspired from QF) | - |
| 85 | Cooperation | require players to work together | New item | New item (inspired from QF) | - |
| 86 | Customization | allow players to customize their in-game objects (e.g.., avatar/vehicle/stuff…) | New item | New item (inspired from Yee2006 & QF) | - |
| 87 | Customization | provide players with a lot of customization options | New item | New item (inspired from Yee2006 & QF) | - |
| 88 | Customization | allow players to personalize their objects/stuff/characters so that those can be unique | New item | New item (inspired from Yee2006 & QF) | - |
| 89 | Destruction | allow players to make explosions | New item | New item (inspired from QF) | - |
| 90 | Destruction | involve destruction | New item | New item (inspired from QF) | - |
| 91 | Destruction | allow players to mess things up | New item | New item (inspired from QF) | - |
| 92 | Graphics | are visually breathtaking | New item | - | - |
| 93 | Graphics | have outstanding graphics | New item | - | - |
| 94 | Graphics | have good graphics, are beautiful | New item | - | - |
| 95 | Story | involve an interesting story | New item | New item (inspired from QF) | - |
| 96 | Story | involve an elaborate story that stimulates my emotions | New item | New item (inspired from QF) | - |
| 97 | Story | involve an immersive narrative | New item | New item (inspired from QF) | - |
| 98 | Strategy | require strategic thinking | New item | New item (inspired from QF) | - |
| 99 | Strategy | require planning ahead and making strategic decisions | New item | New item (inspired from QF) | - |
| 100 | Strategy | require tactical decision making | New item | New item (inspired from QF) | - |

*Note.* Beard: Beard & Wickham, 2016; DeGrove: DeGrove et al., 2016; Ferguson&Olson: Ferguson & Olson, 2013; GAMS: Gaming Motivation Scale, Lafrenière et al., 2012; Ghazali: Ghazali et al., 2019; Hagström: Hagström & Kaldo, 2014; Hamari: Hamari et al., 2019; Hilgard: Hilgard et al., 2013; Hsu: Hsu et al., 2009; Li: Li et al., 2015; MOGQ: Motives for Online Gaming Questionnaire, Demetrovics et al., 2011; Passion: Przybylski et al., 2009; PENS: Player Experience of Need Satisfaction, Ryan et al., 2006; QF: the Gamer Motivation Profile of the Quantic Foundry, https://quanticfoundry.com/; Sherry: Sherry et al., 2006; Trojan: Kahn et al., 2015; Wallenius: Wallenius et al., 2015; Wu: Wu et al., 2010; YEE2006: Yee, 2006; YEE2012: Yee et al., 2012.

**Supplemental Table S3**

*Questions Regarding the COVID-19 Situation and Restrictions (N =* 12,921)

| How much has the coronavirus situation changed how much time you spend playing video games (compared to the times before the pandemic)? |  |
| --- | --- |
| I play much less | 1.5% |
| I play a bit less | 3.1% |
| I play approximately the same | 29.0% |
| I play a bit more | 38.2% |
| I play much more | 28.2% |
| How much has the coronavirus situation changed your gaming habits? Here do not think of your gaming time, but other aspects such as when you play, what types of games you play, with whom you play, or with what motives. |  |
| Not at all | 42.0% |
| A little | 28.0% |
| Moderately | 17.1% |
| Quite a bit | 10.0% |
| Very much | 2.8% |
| How much has the coronavirus situation changed your mood and how you feel in general? |  |
| My mood has worsened considerably | 12.7% |
| My mood has somewhat worsened | 43.1% |
| My mood has not changed | 35.9% |
| My mood has slightly improved | 5.8% |
| My mood has improved considerably | 2.5% |

**Supplemental Table S4**

*Psychometric Examination of the 27 Theoretically Proposed Motivational F*actors

|  | **Item number** |  | **Fit indices and reliability coefficients of the theoretically proposed motivational factors** | | | | |  | **Fit indices and reliability coefficients of the modified motivational factors** | | | |
| --- | --- | --- | --- | --- | --- | --- | --- | --- | --- | --- | --- | --- |
| **Motivational factor** |  | **Item** | **Factor loading** | **RMSEA** | **CFI** | **Cronbach's alpha** | **Highest modification indices (from those > 100)** | **Items removed** | **RMSEA** | **CFI** | **Factor loading** | **Alpha after modification (if a modification happened)** |
| Advancement | 1 | because I like the feeling of continuous advancement | 0.715*** | 0.000 | 1.000 | 0.79 | - | - | 0.000 | 1.000 | 0.715*** | 0.79 |
|  | 2 | because I like to advance in games | 0.850*** |  |  |  | - |  |  |  | 0.850*** |  |
|  | 3 | because I like it when I get to the next level/stage/point in games | 0.690*** |  |  |  | - |  |  |  | 0.690*** |  |
| Amotivation | 4 | I used to have good reasons, but now I am asking myself if I should continue | 0.710*** | 0.000 | 1.000 | 0.81 | - | - | 0.000 | 1.000 | 0.710*** | 0.81 |
|  | 5 | Honestly, I don’t know; I have the impression that I’m wasting my time | 0.727*** |  |  |  | - |  |  |  | 0.727*** |  |
|  | 6 | It is not clear anymore; I sometimes ask myself if it is good for me | 0.865*** |  |  |  | - |  |  |  | 0.865*** |  |
| Autonomy | 7 | because I can determine for myself what I do in games | 0.704*** | 0.079 | 0.969 | 0.81 | 237 (Items 9 and 10) | Item 9 | 0.039 | 0.996 | 0.740*** | 0.78 |
|  | 8 | because I can play the games according to my preferences | 0.678*** |  |  |  | 228 (Items 7 and 8) |  |  |  | 0.696*** |  |
|  | 9 | because they allow me to live memorable experiences | 0.666*** |  |  |  | 211 (Items 7 and 9) |  |  |  |  |  |
|  | 10 | because they provide me with interesting options and choices | 0.717*** |  |  |  |  |  |  |  | 0.631*** |  |
|  | 11 | because I experience a lot of freedom in games | 0.614*** |  |  |  |  |  |  |  | 0.697*** |  |
| Boredom | 12 | because I am bored | 0.792*** | 0.000 | 1.000 | 0.76 | - | - | 0.000 | 1.000 | 0.792*** | 0.76 |
|  | 13 | to pass the time | 0.674*** |  |  |  | - |  |  |  | 0.674*** |  |
|  | 14 | because there is nothing else to do | 0.689*** |  |  |  | - |  |  |  | 0.689*** |  |
| Competence | 15 | because when I perform well, it makes me feel good about myself | 0.766*** | 0.099 | 0.978 | 0.82 | 302 (Items 16 and 15) | Item 17 | 0.000 | 1.000 | 0.797*** | 0.79 |
|  | 16 | because when I'm successful, it boosts my self-esteem | 0.750*** |  |  |  | 302 (Items 18 and 17) |  |  |  | 0.768*** |  |
|  | 17 | because I feel competent when I play | 0.693*** |  |  |  | - |  |  |  | - |  |
|  | 18 | because I feel very capable and effective when playing | 0.722*** |  |  |  | - |  |  |  | 0.672*** |  |
| Competition | 19 | because I like competing with others | 0.761*** | 0.117 | 0.960 | 0.89 | 977 (Items 20 and 19) | Item 20, Item 23 | 0.000 | 1.000 | 0.737*** | 0.82 |
|  | 20 | because competition stimulates me | 0.790*** |  |  |  | 366 (Items 23 and 22) |  |  |  | - |  |
|  | 21 | because I like to win | 0.745*** |  |  |  | 307 (Items 23 and 19) |  |  |  | 0.743*** |  |
|  | 22 | because I like to be better than others | 0.834*** |  |  |  | 136 (Items 23 and 20) |  |  |  | 0.837*** |  |
|  | 23 | because I like to be the best | 0.819*** |  |  |  | 166 (Items 22 and 20) |  |  |  | - |  |
| Completion | 24 | until I get 100% on them, completing everything possible | 0.869*** | 0.000 | 1.000 | 0.80 | - | - | 0.000 | 1.000 | 0.869*** | 0.80 |
|  | 25 | until I unlock all achievements | 0.802*** |  |  |  | - |  |  |  | 0.802*** |  |
|  | 26 | until I master all elements of a game | 0.616*** |  |  |  | - |  |  |  | 0.616*** |  |
| Coping | 27 | because it helps me get my anger out | 0.784*** | 0.148 | 0.948 | 0.75 | 520 (Items 29 and 27) | Item 28 | 0.000 | 1.000 | 0.760*** | 0.75 |
|  | 28 | because it helps me feel less lonely | 0.449*** |  |  |  | 520 (Items 28 and 30) |  |  |  | - |  |
|  | 29 | because it helps me get rid of stress | 0.805*** |  |  |  | 330 (Items 28 and 29) |  |  |  | 0.849*** |  |
|  | 30 | because it helps me get into a better mood | 0.584*** |  |  |  | 330 (Items 30 and 27) |  |  |  | 0.555*** |  |
| Escape | 31 | to avoid thinking about some of your real-life problems or worries | 0.876*** | 0.085 | 0.980 | 0.91 | 198 (Items 32 and 31) | Item 34 | 0.011 | 1.00 | 0.888*** | 0.89 |
|  | 32 | because gaming helps me to forget about daily hassles | 0.817*** |  |  |  | 124 (Items 34 and 32) |  |  |  | 0.832*** |  |
|  | 33 | to forget about unpleasant things or offences | 0.738*** |  |  |  | 127 (Items 35 and 31) |  |  |  | 0.745*** |  |
|  | 34 | because it makes me forget real life | 0.820*** |  |  |  | 551 (Items 35 and 34) |  |  |  | - |  |
|  | 35 | because gaming helps me escape reality | 0.847*** |  |  |  | - |  |  |  | 0.816*** |  |
| Exploration | 36 | because of their ability to surprise me in unexpected ways | 0.614*** | 0.093 | 0.978 | 0.78 | 244 (Items 37 and 36) | Item 36 | 0.000 | 1.000 | - | 0.76 |
|  | 37 | because I like to explore different elements or possibilities of the game | 0.681*** |  |  |  | 244 (Items 39 and 38) |  |  |  | 0.636*** |  |
|  | 38 | because I like to discover unconventional ways to play the game | 0.726*** |  |  |  | 129 (Items 39 and 36) |  |  |  | 0.732*** |  |
|  | 39 | because I like to experiment with different ways to play the game | 0.735*** |  |  |  | - |  |  |  | 0.771*** |  |
| Mechanics | 40 | I like to figure out how specific game elements work in detail | 0.837*** | 0.000 | 1.000 | 0.82 | - | - | 0.000 | 1.000 | 0.837*** | 0.82 |
|  | 41 | because I like to explore the underlying rules of the game | 0.713*** |  |  |  | - |  |  |  | 0.713*** |  |
|  | 42 | because I like to discover/learn the game mechanics thoroughly | 0.787*** |  |  |  | - |  |  |  | 0.787*** |  |
| Fantasy | 43 | because I can do things that I am unable to do or I am not allowed to do in real life | 0.613*** | 0.070 | 0.981 | 0.82 | 158 (Items 46 and 45) | Item 47 | 0.063 | 0.992 | 0.613*** | 0.81 |
|  | 44 | because I can be in another world | 0.851*** |  |  |  | 205 (Items 47 and 46) |  |  |  | 0.865*** |  |
|  | 45 | to be somebody else or somewhere else for a while | 0.795*** |  |  |  | - |  |  |  | 0.789*** |  |
|  | 46 | because I feel immersed in the virtual world | 0.653*** |  |  |  | - |  |  |  | 0.638*** |  |
|  | 47 | because I like to role-play | 0.562*** |  |  |  | - |  |  |  | - |  |
| Financial | 48 | because I have the possibility to earn money | 0.892*** | 0.000 | 1.000 | 0.91 | - | - | 0.000 | 1.000 | 0.892*** | 0.91 |
|  | 49 | because I have the chance to earn some extra income | 0.850 *** |  |  |  | - |  |  |  | 0.850 *** |  |
|  | 50 | because I can make some money | 0.893*** |  |  |  | - |  |  |  | 0.893*** |  |
| Game skills | 51 | because I like to perform to the best of my ability | 0.745*** | 0.000 | 1.000 | 0.86 | - | - | 0.000 | 1.000 | 0.745*** | 0.86 |
|  | 52 | because I like to improve specific gaming skills | 0.740*** |  |  |  | - |  |  |  | 0.740*** |  |
|  | 53 | because I like to continuously improve my own gameplay | 0.824*** |  |  |  | - |  |  |  | 0.824*** |  |
|  | 54 | because I like to practice and master a game | 0.797*** |  |  |  | - |  |  |  | 0.797*** |  |
| Identity | 55 | because playing games is a meaningful activity | 0.771*** | 0.047 | 0.990 | 0.83 | 148 (Items 57 and 55) | - | 0.047 | 0.990 | 0.771*** | 0.83 |
|  | 56 | because it is an extension of me | 0.667*** |  |  |  | - |  |  |  | 0.667*** |  |
|  | 57 | because it is an integral part of my life | 0.763*** |  |  |  | - |  |  |  | 0.763*** |  |
|  | 58 | because it has personal significance to me | 0.730*** |  |  |  | - |  |  |  | 0.730*** |  |
|  | 59 | because this game/gaming is in harmony with the other activities in my life | 0.569*** |  |  |  | - |  |  |  | 0.569*** |  |
| Introjected regulation | 60 | because I must play to feel good about myself | 0.750*** | 0.000 | 1.000 | 0.78 | - | - | 0.000 | 1.000 | 0.750*** | 0.78 |
|  | 61 | because otherwise I would feel bad about myself | 0.735*** |  |  |  | - |  |  |  | 0.735*** |  |
|  | 62 | because I feel that I must play regularly | 0.722*** |  |  |  | - |  |  |  | 0.722*** |  |
| Recreation | 63 | because it is fun | 0.623*** | 0.159 | 0.916 | 0.76 | 767 (Items 65 and 64) | Item 66 | 0.000 | 1.000 | 0.569*** | 0.75 |
|  | 64 | for recreation | 0.781*** |  |  |  | 767 (Items 66 and 63) |  |  |  | 0.810*** |  |
|  | 65 | to relax | 0.756*** |  |  |  | 181 (Items 65 and 63) |  |  |  | 0.767*** |  |
|  | 66 | because playing gives me a lot of pleasure | 0.541*** |  |  |  | 181 (Items 66 and 64) |  |  |  | - |  |
| Skill development | 67 | because gaming sharpens my senses | 0.777*** | 0.002 | 1.000 | 0.90 | - | - | 0.002 | 1.000 | 0.777*** | 0.90 |
|  | 68 | because it improves my skills | 0.842*** |  |  |  | - |  |  |  | 0.842*** |  |
|  | 69 | because it improves my concentration | 0.850*** |  |  |  | - |  |  |  | 0.850*** |  |
|  | 70 | because it improves my coordination skills | 0.834*** |  |  |  | - |  |  |  | 0.834*** |  |
| Social | 71 | because I can get to know new people | 0.784*** | 0.131 | 0.938 | 0.83 | 987 (Items 75 and 72) | Item 75 | 0.083 | 0.986 | 0.800*** | 0.82 |
|  | 72 | because I like playing with others | 0.663*** |  |  |  | 402 (Items 74 and 72) |  |  |  | 0.625*** |  |
|  | 73 | because I feel close to other gamers | 0.734*** |  |  |  | 212 (Items 75 and 71) |  |  |  | 0.742*** |  |
|  | 74 | because I find the relationships I form in games meaningful | 0.770*** |  |  |  | 211 (Items 74 and 73) |  |  |  | 0.775*** |  |
|  | 75 | because games are a uniting interest and common topic to discuss with friends | 0.590*** |  |  |  | - |  |  |  | - |  |
| Status | 76 | for the prestige of being a good player | 0.808*** | 0.000 | 1.000 | 0.83 | - | - | 0.000 | 1.000 | 0.808*** | 0.83 |
|  | 77 | because I gain recognition and esteem from others | 0.820*** |  |  |  | - |  |  |  | 0.820*** |  |
|  | 78 | because others perceive me as skilled | 0.763*** |  |  |  | - |  |  |  | 0.763*** |  |
| Arousal-action | 79 | raise the level of adrenaline | 0.779*** | 0.074 | 0.989 | 0.86 | 150 (Items 80 and 79) | - | 0.074 | 0.989 | 0.779*** | 0.86 |
|  | 80 | keep the players on the edge of their seats | 0.783*** |  |  |  | 150 (Items 82 and 81) |  |  |  | 0.783*** |  |
|  | 81 | raise the level of excitement | 0.848*** |  |  |  | - |  |  |  | 0.848*** |  |
|  | 82 | are intense and full of action | 0.700*** |  |  |  | - |  |  |  | 0.700*** |  |
| Cooperation | 83 | allow players to cooperate with others | 0.902*** | 0.000 | 1.000 | 0.93 | - | - | 0.000 | 1.000 | 0.902*** | 0.93 |
|  | 84 | promote working together in a group | 0.921*** |  |  |  | - |  |  |  | 0.921*** |  |
|  | 85 | require players to work together | 0.878*** |  |  |  | - |  |  |  | 0.878*** |  |
| Customization | 86 | allow players to customize their in-game objects (e.g., avatar/vehicle/stuff…) | 0.855*** | 0.000 | 1.000 | 0.90 | - | - | 0.000 | 1.000 | 0.855*** | 0.90 |
|  | 87 | provide players with a lot of customization options | 0.846*** |  |  |  | - |  |  |  | 0.846*** |  |
|  | 88 | allow players to personalize their objects/stuff/characters so that those can be unique | 0.869*** |  |  |  | - |  |  |  | 0.869*** |  |
| Destruction | 89 | allow players to make explosions | 0.838*** | 0.000 | 1.000 | 0.87 | - | - | 0.000 | 1.000 | 0.838*** | 0.87 |
|  | 90 | involve destruction | 0.895*** |  |  |  | - |  |  |  | 0.895*** |  |
|  | 91 | allow players to mess things up | 0.769*** |  |  |  | - |  |  |  | 0.769*** |  |
| Graphics | 92 | are visually breathtaking | 0.826*** | 0.000 | 1.000 | 0.92 | - | - | 0.000 | 1.000 | 0.826*** | 0.92 |
|  | 93 | have outstanding graphics | 0.915*** |  |  |  | - |  |  |  | 0.915*** |  |
|  | 94 | have good graphics, are beautiful | 0.917*** |  |  |  | - |  |  |  | 0.917*** |  |
| Story | 95 | involve an interesting story | 0.881*** | 0.000 | 1.000 | 0.91 | - | - | 0.000 | 1.000 | 0.881*** | 0.91 |
|  | 96 | involve an elaborate story that stimulates my emotions | 0.862*** |  |  |  | - |  |  |  | 0.862*** |  |
|  | 97 | involve an immersive narrative | 0.893*** |  |  |  | - |  |  |  | 0.893*** |  |
| Strategy | 98 | require strategic thinking | 0.845*** | 0.000 | 1.000 | 0.87 | - | - | 0.000 | 1.000 | 0.845*** | 0.87 |
|  | 99 | require planning ahead and making strategic decisions | 0.858*** |  |  |  | - |  |  |  | 0.858*** |  |
|  | 100 | require tactical decision making | 0.804*** |  |  |  | - |  |  |  | 0.804*** |  |
|  |  |  |  |  |  |  |  |  |  |  |  |  |
| Exploration + Mechanics | 36 | because of their ability to surprise me in unexpected ways | 0.576*** | 0.060 | 0.976 | 0.88 | 286 (Items 39 and 38) | Item 36, Item 38, Item 41 | 0.034 | 0.998 | - | 0.83 |
|  | 37 | because I like to explore different elements or possibilities of the game | 0.696*** |  |  |  | 226 (Items 37 and 36) |  |  |  | 0.678*** |  |
|  | 38 | because I like to discover unconventional ways to play the game | 0.689*** |  |  |  | 223 (Items 62 and 60) |  |  |  | - |  |
|  | 39 | because I like to experiment with different ways to play the game | 0.717*** |  |  |  | 105 (Items 60 and 38) |  |  |  | 0.689*** |  |
|  | 40 | because I like to figure out how specific game elements work in detail | 0.801*** |  |  |  | 101 (Items 60 and 36) |  |  |  | 0.825*** |  |
|  | 41 | because I like to explore the underlying rules of the game | 0.724*** |  |  |  | - |  |  |  | - |  |
|  | 42 | because I like to discover/learn the game mechanics thoroughly | 0.774*** |  |  |  | - |  |  |  | 0.791*** |  |

*Note.* Because of the large size of this table, we report only the CFI and the RMSEA from the available fit indices. When only three items covered a factor, the degree of freedom was 0 and model fit was not informative. RMSEA = root-mean-square error of approximation; CFI = comparative fit index.

****p* < .001

**Supplemental Table S5**

*Descriptive Statistics for the 26 Motives*

|  | *N* | Min | Max | Mean | *SD* | Skewness | | | Kurtosis | |
| --- | --- | --- | --- | --- | --- | --- | --- | --- | --- | --- |
|  |  |  |  |  |  | Statistic | *SE* | Statistic | | *SE* |
| Advancement | 14,232 | 1.00 | 7.00 | 4.94 | 1.48 | -0.550 | 0.021 | -0.349 | | 0.041 |
| Amotivation | 14,248 | 1.00 | 7.00 | 1.83 | 1.21 | 1.834 | 0.021 | 3.211 | | 0.041 |
| Autonomy | 14,202 | 1.00 | 7.00 | 4.46 | 1.49 | -0.295 | 0.021 | -0.611 | | 0.041 |
| Boredom | 14,245 | 1.00 | 7.00 | 3.01 | 1.56 | 0.542 | 0.021 | -0.526 | | 0.041 |
| Competence | 14,235 | 1.00 | 7.00 | 3.40 | 1.63 | 0.287 | 0.021 | -0.835 | | 0.041 |
| Competition | 14,238 | 1.00 | 7.00 | 3.65 | 1.72 | 0.245 | 0.021 | -0.924 | | 0.041 |
| Completion | 14,250 | 1.00 | 7.00 | 4.22 | 1.67 | -0.126 | 0.021 | -0.915 | | 0.041 |
| Coping | 14,229 | 1.00 | 7.00 | 4.04 | 1.63 | 0.022 | 0.021 | -0.888 | | 0.041 |
| Escape | 14,218 | 1.00 | 7.00 | 3.34 | 1.86 | 0.428 | 0.021 | -1.013 | | 0.041 |
| Exploration + Mechanics | 14,188 | 1.00 | 7.00 | 4.22 | 1.53 | -0.138 | 0.021 | -0.717 | | 0.041 |
| Fantasy | 14,233 | 1.00 | 7.00 | 4.25 | 1.70 | -0.118 | 0.021 | -1.014 | | 0.041 |
| Financial | 14,247 | 1.00 | 7.00 | 1.33 | 0.87 | 3.515 | 0.021 | 13.749 | | 0.041 |
| Game skills | 14,193 | 1.00 | 7.00 | 4.22 | 1.62 | -0.151 | 0.021 | -0.826 | | 0.041 |
| Identity | 14,183 | 1.00 | 7.00 | 3.10 | 1.46 | 0.456 | 0.021 | -0.581 | | 0.041 |
| Introjected regulation | 14,250 | 1.00 | 7.00 | 2.07 | 1.26 | 1.401 | 0.021 | 1.612 | | 0.041 |
| Recreation | 14,256 | 1.00 | 7.00 | 6.00 | 1.05 | -1.134 | 0.021 | 0.968 | | 0.041 |
| Skill development | 14,202 | 1.00 | 7.00 | 4.06 | 1.67 | -0.086 | 0.021 | -0.872 | | 0.041 |
| Social | 14,209 | 1.00 | 7.00 | 3.16 | 1.53 | 0.461 | 0.021 | -0.621 | | 0.041 |
| Status | 14,249 | 1.00 | 7.00 | 2.48 | 1.53 | 0.982 | 0.021 | 0.129 | | 0.041 |
| Arousal-action | 14,322 | 1.00 | 7.00 | 4.88 | 1.43 | -0.462 | 0.020 | -0.385 | | 0.041 |
| Cooperation | 14,330 | 1.00 | 7.00 | 4.37 | 1.86 | -0.252 | 0.020 | -1.031 | | 0.041 |
| Customization | 14,338 | 1.00 | 7.00 | 4.86 | 1.69 | -0.488 | 0.020 | -0.684 | | 0.041 |
| Destruction | 14,326 | 1.00 | 7.00 | 3.43 | 1.70 | 0.357 | 0.020 | -0.780 | | 0.041 |
| Graphics | 14,324 | 1.00 | 7.00 | 5.26 | 1.58 | -0.722 | 0.020 | -0.274 | | 0.041 |
| Story | 14,337 | 1.00 | 7.00 | 5.61 | 1.60 | -1.122 | 0.020 | 0.387 | | 0.041 |
| Strategy | 14,334 | 1.00 | 7.00 | 5.06 | 1.46 | -0.517 | 0.020 | -0.383 | | 0.041 |

*Note.* Min = minimum value; Max = maximum value.

**Supplemental Table S6**

*Zero-Order Correlations Between the 26 Motivational F*actors

|  | 1 | 2 | 3 | 4 | 5 | 6 | 7 | 8 | 9 | 10 | 11 | 12 | 13 | 14 | 15 | 16 | 17 | 18 | 19 | 20 | 21 | 22 | 23 | 24 | 25 | 26 |
| --- | --- | --- | --- | --- | --- | --- | --- | --- | --- | --- | --- | --- | --- | --- | --- | --- | --- | --- | --- | --- | --- | --- | --- | --- | --- | --- |
| 1. Advancement | 1 |  |  |  |  |  |  |  |  |  |  |  |  |  |  |  |  |  |  |  |  |  |  |  |  |  |
| 1. Amotivation | -.056^**^ | 1 |  |  |  |  |  |  |  |  |  |  |  |  |  |  |  |  |  |  |  |  |  |  |  |  |
| 1. Autonomy | .600^**^ | -.014 | 1 |  |  |  |  |  |  |  |  |  |  |  |  |  |  |  |  |  |  |  |  |  |  |  |
| 1. Boredom | .053^**^ | .327^**^ | .109^**^ | 1 |  |  |  |  |  |  |  |  |  |  |  |  |  |  |  |  |  |  |  |  |  |  |
| 1. Competence | .530^**^ | .088^**^ | .522^**^ | .162^**^ | 1 |  |  |  |  |  |  |  |  |  |  |  |  |  |  |  |  |  |  |  |  |  |
| 1. Competition | .393^**^ | .109^**^ | .253^**^ | .149^**^ | .532^**^ | 1 |  |  |  |  |  |  |  |  |  |  |  |  |  |  |  |  |  |  |  |  |
| 1. Completion | .665^**^ | -.062^**^ | .497^**^ | .026^**^ | .461^**^ | .365^**^ | 1 |  |  |  |  |  |  |  |  |  |  |  |  |  |  |  |  |  |  |  |
| 1. Coping | .331^**^ | .031^**^ | .472^**^ | .203^**^ | .444^**^ | .209^**^ | .255^**^ | 1 |  |  |  |  |  |  |  |  |  |  |  |  |  |  |  |  |  |  |
| 1. Escape | .233^**^ | .147^**^ | .430^**^ | .239^**^ | .411^**^ | .106^**^ | .172^**^ | .627^**^ | 1 |  |  |  |  |  |  |  |  |  |  |  |  |  |  |  |  |  |
| 1. Exploration + Mechanics | .665^**^ | -.038^**^ | .617^**^ | .031^**^ | .467^**^ | .290^**^ | .661^**^ | .292^**^ | .195^**^ | 1 |  |  |  |  |  |  |  |  |  |  |  |  |  |  |  |  |
| 1. Fantasy | .435^**^ | .019^*^ | .695^**^ | .140^**^ | .439^**^ | .123^**^ | .340^**^ | .519^**^ | .618^**^ | .410^**^ | 1 |  |  |  |  |  |  |  |  |  |  |  |  |  |  |  |
| 1. Financial | .110^**^ | .133^**^ | .119^**^ | .082^**^ | .254^**^ | .243^**^ | .138^**^ | .108^**^ | .103^**^ | .146^**^ | .058^**^ | 1 |  |  |  |  |  |  |  |  |  |  |  |  |  |  |
| 1. Game skills | .694^**^ | -.032^**^ | .524^**^ | .049^**^ | .652^**^ | .579^**^ | .675^**^ | .329^**^ | .208^**^ | .673^**^ | .326^**^ | .232^**^ | 1 |  |  |  |  |  |  |  |  |  |  |  |  |  |
| 1. Identity | .476^**^ | -.021^*^ | .596^**^ | .094^**^ | .612^**^ | .288^**^ | .395^**^ | .469^**^ | .458^**^ | .474^**^ | .568^**^ | .223^**^ | .505^**^ | 1 |  |  |  |  |  |  |  |  |  |  |  |  |
| 1. Introjected regulation | .261^**^ | .195^**^ | .340^**^ | .267^**^ | .482^**^ | .269^**^ | .230^**^ | .401^**^ | .476^**^ | .223^**^ | .394^**^ | .236^**^ | .292^**^ | .554^**^ | 1 |  |  |  |  |  |  |  |  |  |  |  |
| 1. Recreation | .356^**^ | -.185^**^ | .383^**^ | .048^**^ | .170^**^ | .083^**^ | .232^**^ | .364^**^ | .214^**^ | .280^**^ | .347^**^ | -.084^**^ | .228^**^ | .274^**^ | .114^**^ | 1 |  |  |  |  |  |  |  |  |  |  |
| 1. Skill development | .516^**^ | -.055^**^ | .498^**^ | .043^**^ | .540^**^ | .362^**^ | .455^**^ | .382^**^ | .240^**^ | .551^**^ | .347^**^ | .208^**^ | .672^**^ | .501^**^ | .261^**^ | .237^**^ | 1 |  |  |  |  |  |  |  |  |  |
| 1. Social | .317^**^ | .089^**^ | .318^**^ | .130^**^ | .477^**^ | .407^**^ | .279^**^ | .284^**^ | .217^**^ | .354^**^ | .217^**^ | .291^**^ | .461^**^ | .430^**^ | .281^**^ | .115^**^ | .436^**^ | 1 |  |  |  |  |  |  |  |  |
| 1. Status | .373^**^ | .139^**^ | .335^**^ | .171^**^ | .692^**^ | .617^**^ | .362^**^ | .301^**^ | .275^**^ | .329^**^ | .249^**^ | .367^**^ | .557^**^ | .466^**^ | .423^**^ | .035^**^ | .432^**^ | .555^**^ | 1 |  |  |  |  |  |  |  |
| 1. Arousal-action | .436^**^ | -.013 | .405^**^ | .145^**^ | .419^**^ | .414^**^ | .369^**^ | .376^**^ | .232^**^ | .372^**^ | .325^**^ | .134^**^ | .485^**^ | .341^**^ | .258^**^ | .281^**^ | .422^**^ | .336^**^ | .354^**^ | 1 |  |  |  |  |  |  |
| 1. Cooperation | .249^**^ | .062^**^ | .186^**^ | .070^**^ | .321^**^ | .393^**^ | .212^**^ | .156^**^ | .061^**^ | .279^**^ | .059^**^ | .185^**^ | .401^**^ | .209^**^ | .122^**^ | .101^**^ | .340^**^ | .663^**^ | .399^**^ | .396^**^ | 1 |  |  |  |  |  |
| 1. Customization | .403^**^ | -.017^*^ | .440^**^ | .093^**^ | .286^**^ | .147^**^ | .368^**^ | .268^**^ | .229^**^ | .381^**^ | .384^**^ | .048^**^ | .332^**^ | .298^**^ | .172^**^ | .215^**^ | .281^**^ | .235^**^ | .203^**^ | .356^**^ | .231^**^ | 1 |  |  |  |  |
| 1. Destruction | .260^**^ | .080^**^ | .361^**^ | .234^**^ | .310^**^ | .270^**^ | .231^**^ | .336^**^ | .263^**^ | .255^**^ | .365^**^ | .140^**^ | .266^**^ | .275^**^ | .298^**^ | .135^**^ | .251^**^ | .227^**^ | .279^**^ | .530^**^ | .230^**^ | .343^**^ | 1 |  |  |  |
| 1. Graphics | .250^**^ | -.058^**^ | .239^**^ | .061^**^ | .171^**^ | .150^**^ | .243^**^ | .181^**^ | .121^**^ | .196^**^ | .229^**^ | .018^*^ | .214^**^ | .146^**^ | .114^**^ | .233^**^ | .194^**^ | .086^**^ | .129^**^ | .381^**^ | .130^**^ | .377^**^ | .290^**^ | 1 |  |  |
| 1. Story | .328^**^ | -.078^**^ | .394^**^ | .003 | .127^**^ | -.047^**^ | .266^**^ | .215^**^ | .195^**^ | .326^**^ | .397^**^ | -.080^**^ | .148^**^ | .277^**^ | .067^**^ | .309^**^ | .176^**^ | .054^**^ | -.013 | .255^**^ | .030^**^ | .340^**^ | .144^**^ | .313^**^ | 1 |  |
| 1. Strategy | .335^**^ | .011 | .302^**^ | -.004 | .243^**^ | .247^**^ | .318^**^ | .155^**^ | .080^**^ | .401^**^ | .163^**^ | .082^**^ | .372^**^ | .201^**^ | .062^**^ | .192^**^ | .348^**^ | .271^**^ | .201^**^ | .331^**^ | .416^**^ | .258^**^ | .171^**^ | .147^**^ | .236^**^ | 1 |

**p* < .05. ***p* < .01.

**Supplemental Table S7**

*Zero-Order Correlations Between the Six Higher Order Motivational Factors and the Twelve Game G*enres

|  | Mastery | Immersion/Escapism | Competition | Stimulation | Social | Habit/Boredom |
| --- | --- | --- | --- | --- | --- | --- |
| Shooter games, FPS, TPS | -.021^*^ | -.054^**^ | .133^**^ | .139^**^ | .190^**^ | .018^*^ |
| Battle royale | -.049^**^ | -.086^**^ | .171^**^ | .057^**^ | .195^**^ | .063^**^ |
| MOBA | .021^*^ | .038^**^ | .179^**^ | -.032^**^ | .201^**^ | .126^**^ |
| Auto chess/auto battler games | -.020^*^ | -.031^**^ | .037^**^ | -.035^**^ | .016 | .049^**^ |
| Open world action-adventure games | .046^**^ | .071^**^ | -.177^**^ | .094^**^ | -.170^**^ | -.069^**^ |
| Role-playing games | .111^**^ | .145^**^ | -.251^**^ | -.038^**^ | -.250^**^ | -.157^**^ |
| MMORPG | .042^**^ | .050^**^ | -.004 | -.062^**^ | .063^**^ | .013 |
| Strategy games, RTS, TBS | -.006 | -.002 | -.111^**^ | -.108^**^ | -.071^**^ | -.010 |
| Card games | .009 | .004 | -.007 | -.072^**^ | -.031^**^ | .010 |
| Sport games | -.089^**^ | -.110^**^ | .051^**^ | -.013 | -.080^**^ | .019^*^ |
| Simulators | -.038^**^ | -.010 | -.028^**^ | -.082^**^ | -.075^**^ | -.007 |
| Other | -.041^**^ | -.035^**^ | -.082^**^ | -.128^**^ | -.144^**^ | -.018^*^ |

*Note.* FPS = first-person shooter; TPS = third-person shooter; MOBA = multiplayer online battle arena; MMORPG = massively multiplayer online role-playing game; RTS = real-time strategy; TBS = turn-based strategy.

**p* < .05. ***p* < .01.

**Supplemental Table S8**

*Zero-Order Correlation Coefficients of the Variables Included in the Multiple Indicator Multiple Cause Model (N*=12,817–14,549)

|  | Gaming motives | | | | | |
| --- | --- | --- | --- | --- | --- | --- |
| Predictors | Mastery | Immersion/Escapism | Competition | Stimulation | Social | Habit/Boredom |
| Self-esteem | .041* | -.251* | -.016 | .025 | .043* | -.230* |
| Positive affect | .256* | -.127* | .115* | .159* | .216* | -.273* |
| Negative affect | .031* | .373* | .134* | .123* | -.010 | .322* |
| Sociability | .052* | -.155* | .132* | .081* | .346* | -.025 |
| Competitiveness | .145* | -.148* | .454* | .197* | .322* | -.006 |
| Perceived stress | -.033* | .336* | .047* | .038* | -.058* | .309* |
| Sensation seeking | .222* | .034* | .182* | .252* | .272* | -.026 |
| Age | -.104* | -.133* | -.278* | -.167* | -.315* | -.188* |
| Gender | .039* | .115* | -.061* | -.033* | -.102* | -.078* |

*Note.* After Bonferroni correction, *p* < .001 is regarded as significant. Gender: males were coded as 0, females as 1.

**p* < .001.

**Supplemental Table S9**

*Predictive effects in the mediation model (N=14,740)*

| Predictor variables | Outcome variables | | | | | | | |
| --- | --- | --- | --- | --- | --- | --- | --- | --- |
|  | Mastery  β (S.E.) | Immersion/  Escapism  β (S.E.) | Competition  β (S.E.) | Stimulation  β (S.E.) | Social  β (S.E.) | Habit/ Boredom  β (S.E.) | Gaming disorder symptoms  β (S.E.) | Gaming time  β (S.E.) |
| Gender | .042* (.009) | .067* (.009) | -.080* (.009) | -.067* (.012) | -.095* (.010) | -.186* (.014) | -.020 (.008) | -.027 (.009) |
| Age | -.090* (.010) | -.052* (.009) | -.264* (.009) | -.157* (.013) | -.351* (.009) | -.146* (.017) | .016 (.009) | -.098* (.009) |
| Depression symptoms | -.020 (.010) | .389* (.009) | .079* (.010) | .035 (.012) | -.050* (.010) | .415* (.013) | .180* (.013) | -.063* (.014) |
| Mastery |  |  |  |  |  |  | -.003 (.015) | .030 (.018) |
| Immersion/Escapism |  |  |  |  |  |  | .228* (.014) | .240* (.014) |
| Competition |  |  |  |  |  |  | .199* (.015) | .121* (.018) |
| Stimulation |  |  |  |  |  |  | -.006 (.012) | -.092* (.013) |
| Social |  |  |  |  |  |  | -.032 (.012) | .170* (.014) |
| Habit/Boredom |  |  |  |  |  |  | .216* (.025) | .073 (.031) |
| Explained variance (R^2^) | 0.9% | 17.5% | 8.8% | 3.2% | 12.9% | 22.6% | 28.5% | 18.0% |

Note. β (S.E.): Standardized regression coefficient with the related standard error value. Level of significance: **p <* .001.

**Supplemental Table S10**

*Bivariate Correlations Between Gender, Age, Depression Symptoms, Gaming Motives, Gaming Disorder (GD) Symptoms and Gaming Time (N*=12,867–14,537)

|  | GD symptoms | Gaming time | Mastery | Immersion/Escapism | Competition | Stimulation | Social | Habit/Boredom |
| --- | --- | --- | --- | --- | --- | --- | --- | --- |
| Gender | .005 | -.033* | .039* | .115* | -.061* | -.033* | -.102* | -.078* |
| Age | -.140* | -.212* | -.104* | -.133* | -.278* | -.167* | -.315* | -.188* |
| Depression symptoms | .378* | .087* | .020 | .371* | .111* | .085* | .002 | .331* |
| Mastery | .125* | .204* |  |  |  |  |  |  |
| Immersion/Escapism | .350* | .272* |  |  |  |  |  |  |
| Competition | .289* | .304* |  |  |  |  |  |  |
| Stimulation | .135* | .133* |  |  |  |  |  |  |
| Social | .135* | .279* |  |  |  |  |  |  |
| Habit/Boredom | .293* | .120* |  |  |  |  |  |  |

*Note.* Gender was coded as 0=male, 1=female. After Bonferroni correction, *p* < .0025 is regarded as significant.

**p* < .001.

**References**

Beard, C. L., & Wickham, R. E. (2016). Gaming-contingent self-worth, gaming motivation, and Internet Gaming Disorder. *Computers in* *Human Behavior, 61*, 507-515.

De Grove, F., Cauberghe, V., & Van Looy, J. (2016). Development and validation of an instrument for measuring individual motives for playing digital games. *Media Psychology, 19*(1), 101-125.

Demetrovics, Z., Urbán, R., Nagygyörgy, K., Farkas, J., Zilahy, D., Mervó, B., Reindl, A., Ágoston, C., Kertész, A., & Harmath, E. (2011). Why do you play? The development of the Motives for Online Gaming Questionnaire (MOGQ). *Behavior Research Methods, 43*(3), 814-825.

Ferguson, C. J., & Olson, C. K. (2013). Friends, fun, frustration and fantasy: Child motivations for video game play. *Motivation and Emotion, 37*(1), 154-164.

Ghazali, E., Mutum, D. S., & Woon, M. Y. (2019). Exploring player behavior and motivations to continue playing Pokémon GO. *Information Technology & People, 32*(3), 646–667.

Hagström, D., & Kaldo, V. (2014). Escapism among players of MMORPGs—conceptual clarification, its relation to mental health factors, and development of a new measure. *Cyberpsychology, Behavior, and Social Networking, 17*(1), 19-25.

Hamari, J., Malik, A., Koski, J., & Johri, A. (2019). Uses and gratifications of Pokémon Go: why do people play mobile location-based augmented reality games? *International Journal of Human–Computer Interaction, 35*(9), 804-819.

Hilgard, J., Engelhardt, C. R., & Bartholow, B. D. (2013). Individual differences in motives, preferences, and pathology in video games: the gaming attitudes, motives, and experiences scales (GAMES). *Frontiers in Psychology*, 4, 608.

Hsu, S. H., Wen, M. H., & Wu, M. C. (2009). Exploring user experiences as predictors of MMORPG addiction. *Computers & Education, 53*(3), 990-999.

Kahn, A. S., Shen, C., Lu, L., Ratan, R. A., Coary, S., Hou, J., Meng, J., Osborn, J, & Williams, D. (2015). The Trojan Player Typology: A cross-genre, cross-cultural, behaviorally validated scale of video game play motivations. *Computers in Human Behavior*, 49, 354-361.

Lafrenière, M. A. K., Verner-Filion, J., & Vallerand, R. J. (2012). Development and validation of the Gaming Motivation Scale (GAMS). *Personality and Individual Differences, 53*(7), 827-831.

Li, H., Liu, Y., Xu, X., Heikkilä, J., & Van Der Heijden, H. (2015). Modeling hedonic is continuance through the uses and gratifications theory: An empirical study in online games. *Computers in Human Behavior*, 48, 261-272.

Przybylski, A. K., Weinstein, N., Ryan, R. M., & Rigby, C. S. (2009). Having to versus wanting to play: Background and consequences of harmonious versus obsessive engagement in video games. *CyberPsychology & Behavior, 12*(5), 485-492.

Ryan, R. M., Rigby, C. S., & Przybylski, A. (2006). The motivational pull of video games: A self-determination theory approach. *Motivation and Emotion, 30*(4), 344-360.

Sherry, J. L., Lucas, K., Greenberg, B. S., & Lachlan, K. (2006). Video Game Uses and Gratifications as Predictors of Use and Game Preference. In P. Vorderer & J. Bryant (Eds.), *Playing video games: Motives, responses, and consequences* (pp. 213-224). Lawrence Erlbaum Associates Publishers.

Vallerand, R. J., Blanchard, C., Mageau, G. A., Koestner, R., Ratelle, C., Léonard, M., Gagné, M., & Marsolais, J. (2003). Les passions de l'ame: on obsessive and harmonious passion. *Journal of Personality and Social Psychology, 85*(4), 756-767.

Wallenius, M., Rimpelä, A., Punamäki, R. L., & Lintonen, T. (2009). Digital game playing motives among adolescents: Relations to parent–child communication, school performance, sleeping habits, and perceived health. *Journal of Applied Developmental Psychology, 30*(4), 463-474.

Wu, J. H., Wang, S. C., & Tsai, H. H. (2010). Falling in love with online games: The uses and gratifications perspective. *Computers in Human Behavior, 26*(6), 1862-1871.

Yee, N. (2006). Motivations for play in online games. *CyberPsychology & Behavior, 9*(6), 772-775.

Yee, N., Ducheneaut, N., & Nelson, L. (2012). Online gaming motivations scale: development and validation. In *Proceedings of the SIGCHI Conference on Human Factors in Computing Systems* (pp. 2803-2806).
